# Optimizing use of data and evidence-related tools to complement routine surveillance systems for tuberculosis program planning

**DOI:** 10.3389/ftubr.2025.1622167

**Published:** 2025-07-17

**Authors:** Rachel M. Fiorillo, Eveline Klinkenberg, Christina Braccio, Daniel Chin, Stavia Turyahabwe, Moses Arinaitwe, Razia Fatima, Taye Letta, Binh Hoa Nguyen, Aiban Ronoh, Charalambos Sismanidis, Nnamdi B. Nwaneri, Kenneth G. Castro, Kathy Fiekert, Fukushi Morishita, Suvanand Sahu, Sevim Ahmedov, Harry Hausler, Anand Date, Jennifer B. Harris

**Affiliations:** ^1^Infectious Disease Department, CDC Foundation, Atlanta, GA, United States; ^2^Connect TB, The Hague, Netherlands; ^3^Division of Global HIV and Tuberculosis, U.S. Centers for Disease Control and Prevention, Atlanta, GA, United States; ^4^Global Health, Gates Foundation, Seattle, WA, United States; ^5^Tuberculosis/Leprosy Control Division, Ministry of Health, Kampala, Uganda; ^6^KNCV TB Plus, The Hague, Netherlands; ^7^United Nations Office for Project Services, Copenhagen, Denmark; ^8^National Tuberculosis, Leprosy, and Other Lung Disease Prevention and Control Program, Ethiopian Ministry of Health, Addis Ababa, Ethiopia; ^9^Vietnam National Tuberculosis Control Program, Ministry of Health, Hanoi, Vietnam; ^10^Division of Tuberculosis and Lung Health, Ministry of Health, Nairobi, Kenya; ^11^Global TB Programme, World Health Organization, Geneva, Switzerland; ^12^The Global Fund to Fight AIDS, Tuberculosis and Malaria, Geneva, Switzerland; ^13^Hubert Department of Global Health and Department of Epidemiology, Emory University Rollins School of Public Health, Atlanta, GA, United States; ^14^Department of Medicine, Emory University School of Medicine, Atlanta, GA, United States; ^15^United States Agency for International Development, Washington, DC, United States; ^16^Integrated Communicable Disease Unit, World Health Organization Regional Office for the Western Pacific, Manila, Philippines; ^17^Stop TB Partnership, Geneva, Switzerland; ^18^TB HIV Care, Cape Town, South Africa; ^19^Department of Family Medicine, University of Pretoria, Pretoria, South Africa

**Keywords:** tuberculosis supplemental data, data utilization, strategic planning, program planning, tuberculosis care cascade, data tools

## Abstract

**Background:**

Challenges and gaps with routine tuberculosis (TB) data and surveillance systems are well-known. To address them, numerous TB data and evidence-related tools (e.g. surveys, assessments) have been developed to help countries collect, analyze, and use TB-related data. The “TB Data Optimization Project” aimed to assess the use and usefulness of these tools and propose best practices.

**Methods:**

Phase one of this mixed-methods project included structured key informant interviews (KIIs) with TB data experts, literature review, and mapping tools' indicators and metrics. Phase two consisted of case studies in five countries (Ethiopia, Kenya, Pakistan, Uganda, and Vietnam). Structured KIIs and a use case discussion were conducted with TB program staff and partners in each country, and TB-related documents were reviewed. Phase three was an online survey for national TB programs. Qualitative data were recorded, transcribed, coded, and inductively analyzed. Quantitative data were analyzed descriptively. Findings were triangulated and summarized into key themes.

**Results:**

Seventy-two KIIs were conducted, 42 countries completed surveys, and 212 documents were reviewed. Six key themes emerged: usefulness, opportunities and challenges with planning and implementation, technical assistance and financial support, timing and coordination, motivating factors for implementation, and the role of tools in relation to routine data systems. The tools provide critical information and most were considered worth the investment. Challenges include suboptimal implementation of the recommendations from the tools, poor timing and coordination of implementation, and insufficient capacity building. A set of best practices was developed.

**Conclusion:**

While the long-term goal is to strengthen and integrate routine data systems, TB data tools currently play an important role in filling gaps. Findings from this project provide considerations for optimal use of TB data tools; however, there is still need for further guidance on selecting the most critical tools to fill gaps during TB programmatic and strategic planning.

## 1 Introduction

High quality tuberculosis (TB) data is crucial for planning and implementing effective TB programs and monitoring progress toward country and global programmatic targets (1). However, routine TB program and surveillance data do not capture all desired information for TB program planning and decision making. This is especially true in many high TB burden countries, where challenges with routine data systems often exist, such as limited system coverage, poor data quality, noncompliance with reporting mandates, fragmentation of data, and limited progress with transitioning to high quality digital data systems (2, 3). In the past two decades, numerous surveys, assessments, and monitoring and evaluation tools have been developed to supplement routine TB surveillance systems and assist countries in the collection, analysis, and utilization of TB-related data. Examples of these TB data tools include TB prevalence surveys, TB patient cost surveys, patient pathway analyses, and diagnostic network optimization tools (4–7). In 2021, the “Compendium of data and evidence-related tools for use in TB planning and programming” (*i.e.*, the “compendium”) was published, which contains the profile for 15 such TB data tools. Each profile in the compendium provides an overview of the tool, including information such as its objectives, key metrics assessed, recommended frequency and average durations of implementation, estimated cost, its limitations, and any pre-requisites for implementation (8).

Questions on whether countries need to implement all these TB data tools and how to implement them in an efficient manner have been raised by National TB Programs (NTPs) as well as technical partners and donors. Additionally, implementation of these TB data tools can be time consuming, often requires specific technical expertise, and may require substantial funding (8). The “TB Data Optimization Project” systematically assessed the use and usefulness of TB data tools and the potential for streamlining them. Findings were synthesized into a set of best practices for the optimization of TB data generation, review, and analysis. This paper summarizes the key findings from the TB Data Optimization Project.

## 2 Methods

The TB Data Optimization Project was a mixed-methods assessment implemented from January 2021 to August 2023 by a project team from the U.S. Centers for Disease Control and Prevention (CDC) and the CDC Foundation, with funding from the Gates Foundation.

A steering committee comprising 10 TB data experts from global TB technical and funding partners, and one Ministry of Health was established to provide input and expertise into the project design, implementation, and interpretation of findings. This committee met seven times virtually throughout the course of the project, with periodic feedback provided between meetings.

### 2.1 TB data tools that are supplemental to routine data and surveillance systems

The compendium (8) served as the starting point for development of a list of TB data tools on which to focus. Additional tools were added after discussions with technical partners active in the global TB community. The final list of focus tools included:

1. TB prevalence surveys (7)
2. Anti-TB drug resistance surveys (9)
3. Inventory studies (10)
4. Private sector drug sales analysis (11)
5. Surveys of costs faced by households affected by TB (previously known as TB patient cost surveys) (6)
6. TB service delivery costing studies (Value TB) (12, 13)
7. OneHealth tool for TB budgeting (14)
8. TB diagnostic network assessment (15)
9. Diagnostic network optimization (16)
10. TB care cascade analysis (17)
11. Mapping and analysis for tailored disease control and health system strengthening (MATCH) (18)
12. Patient pathway analysis (5)
13. People-centered framework (19, 20)
14. Epidemiological reviews including standards and benchmarks assessment (*i.e.*, TB epidemiological reviews) (21)
15. Quality of TB services assessment (22)
16. Epidemiological modeling (23)
17. Screen-TB (24)

**Figure 1:**
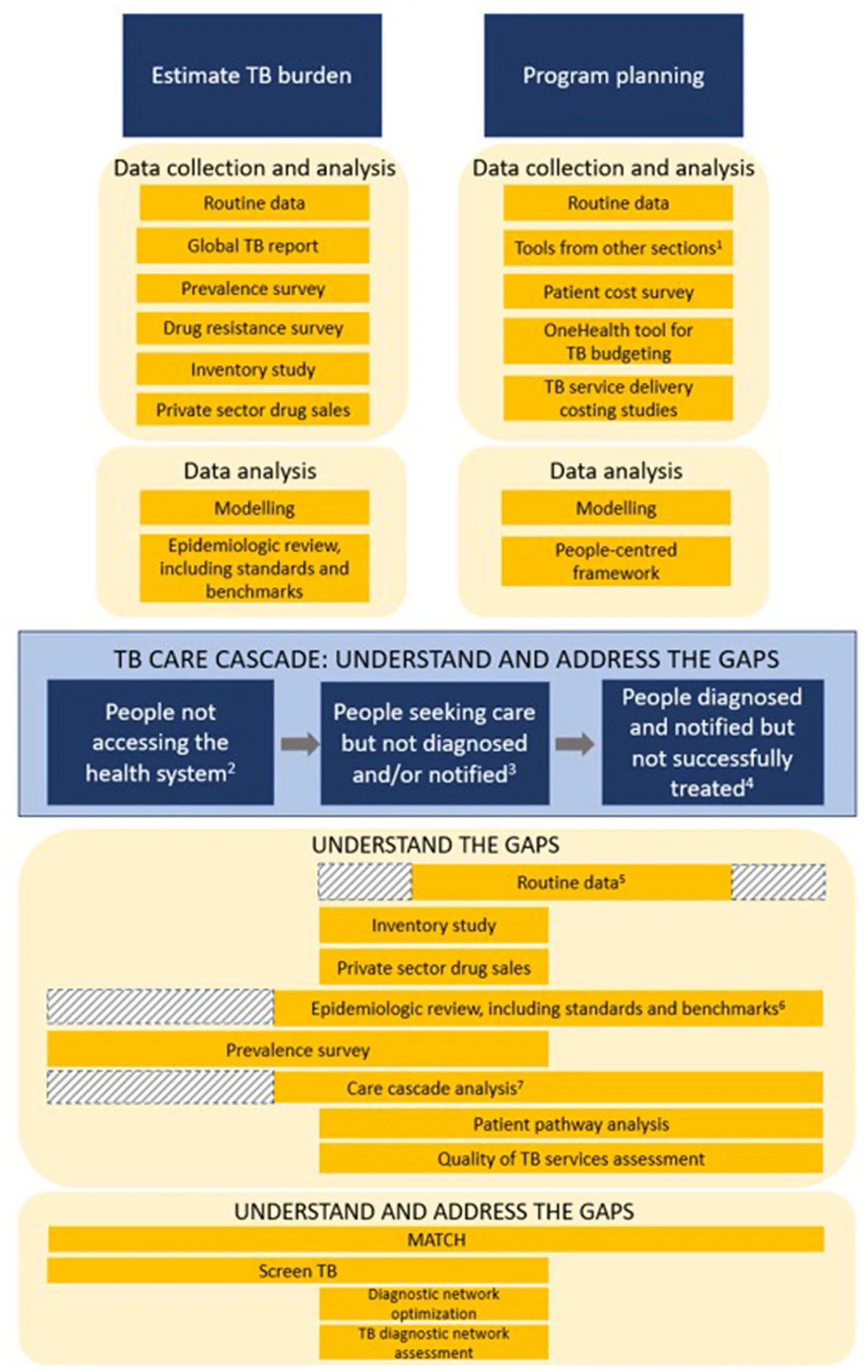
Mapping the use of TB data and TB data tools for TB program planning and evaluation–TB Data Optimization Project, 2021–2023. ^1^Tools from other sections = TB data tools listed under the “Estimate TB burden” and “TB care cascade” sections. ^2^People not accessing the health system = people with TB infection with high risk for disease, people with asymptomatic disease but not seeking care, and people with symptomatic disease but not seeking care. ^3^People seeking care but not diagnosed and/or notified = people presenting to health facilities but not diagnosed, people diagnosed by non-NTP facilities but not notified, and people diagnosed by NTP facilities but not notified. ^4^People diagnosed and notified but not successfully treated = people diagnosed but not started on treatment, people notified but not successfully treated, and people who are successfully treated but not relapse free. ^5^First shaded area = routine data systems in some countries provide TB screening data for TB contacts and/or people presenting for care at health facilities, but in other countries routine TB program data only starts documenting patients after they've been identified as having presumptive TB; Second shaded area = routine data on people diagnosed and notified but not successfully treated may not always be available. ^6^Shaded area = if there is available data from a prevalence survey or another source that looks at people not accessing the health system, then that data may be reviewed and incorporated into an epidemiological review. ^7^Shaded area = unless data from a prevalence survey or another source is available, countries may not have data on people with TB who did not access the health system to use in care cascade analyses.

The 17 TB data tools were mapped into three areas based on where the tool can be used to support national TB programs with: (1) estimating the TB burden, (2) program planning, and (3) understanding the TB care cascade (Figure 1). The care cascade in Figure 1 is modeled after the care cascade used in the People-Centered Framework to align with an existing framework used by countries and partners (19, 20). TB prevention was not included in the figure as none of the tools in the compendium or recommended by partners at the time the project began aimed to assess issues related to TB prevention.

### 2.2 Overview of project design

**Figure 2:**
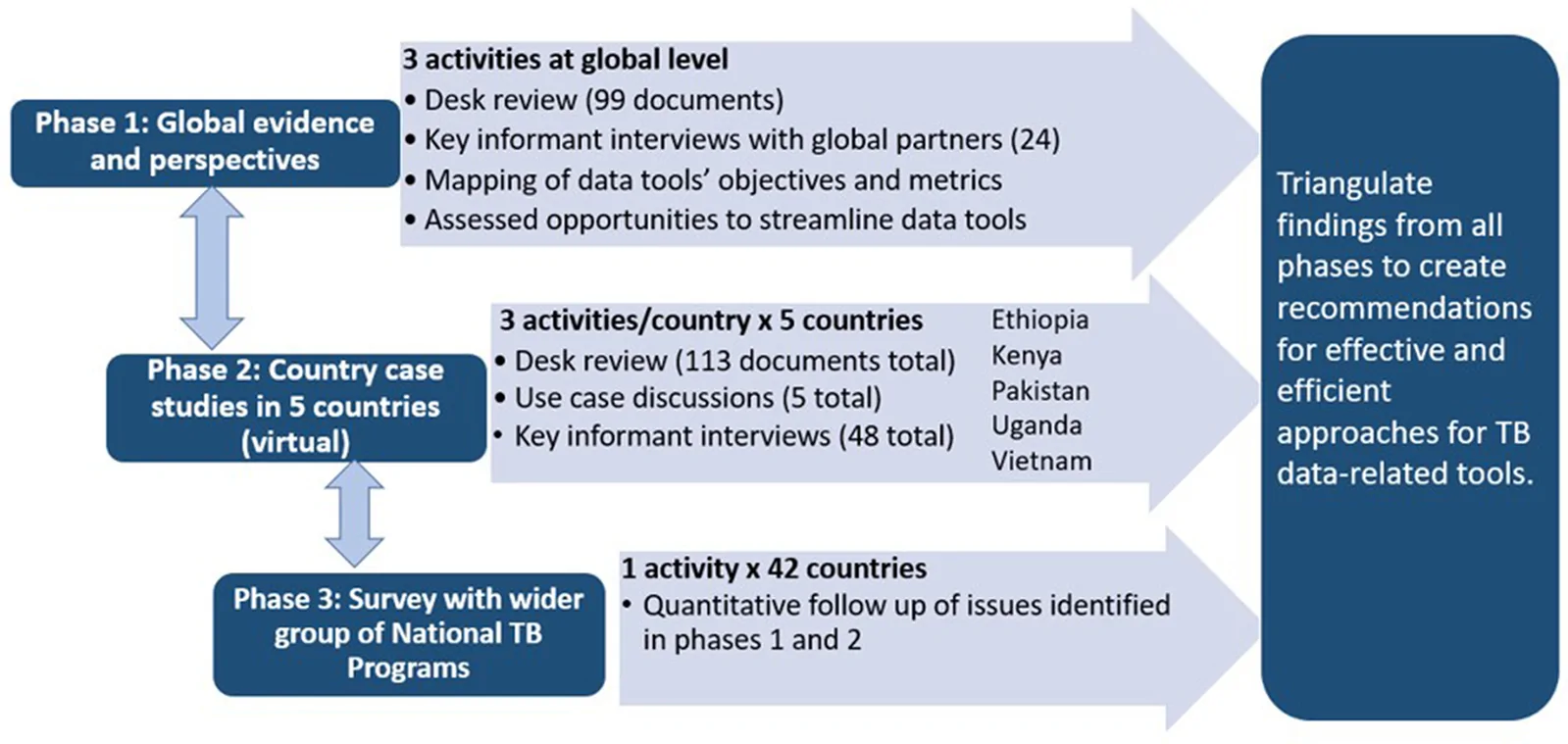
The three phases of the TB data optimization project, 2021–2023.

The project used mixed methods and consisted of three phases which were implemented from January 2021 to August 2023 (Figure 2).

#### 2.2.1 Phase 1: global evidence and perspectives (February 2021 to September 2021)

The first phase consisted of a desk review, indicator mapping, assessment of opportunities to streamline tools, and structured key informant interviews (KIIs) with global technical and funding partners. The desk review was conducted to better understand the tools' methodologies and the impact of these tools on TB programs and policy. Indicators and metrics from TB data tools were mapped and aligned to characterize commonalities and used to investigate potential streamlining of tools and approaches. KIIs were conducted to hear TB data experts' perception on the use and usefulness of these TB data tools.

#### 2.2.2 Phase 2: country case studies in five countries (August 2021–February 2023)

The second phase consisted of country case studies in five purposely selected high TB burden countries: Ethiopia, Kenya, Pakistan, Uganda, and Vietnam. Two other countries were approached but were not able to participate due to competing priorities. Country selection was based on a country's prior experience with TB data tools (all countries had implemented a minimum of nine tools), geographic variety, variation regarding use of electronic and paper-based routine TB data systems, and willingness of the MOH to engage in this project. In each country, we commenced with a desk review of existing evidence (*e.g.*, reports, publications) related to TB data tools the country had previously implemented and reviewed strategic planning documents and funding applications to understand the use of the tools' findings for program planning and monitoring. A use case discussion with NTP staff and in-country partners was conducted in each country to better understand how TB data tools have helped the NTP and TB partners to: (a) estimate the burden of TB in their country; (b) understand and address gaps in the TB care cascade; and (c) develop annual program and national strategic plans. Individual KIIs collected perceptions on the use and usefulness of various TB data for program planning and decision making as well as successes and challenges encountered when planning and implementing TB data tools.

#### 2.2.3 Phase 3: national TB program survey (August 2022–June 2023)

The third phase consisted of an online quantitative survey for NTP managers or their designees. The questions were developed from information gathered during phase one and two to seek similar information from a wider range of countries.

### 2.3 Sampling and data collection

#### 2.3.1 Desk reviews

Documents targeted for the global review were obtained through an English-language search of peer-reviewed journals, websites, and requests to tool developers using a range of general terms related to TB data tools as well as the names of each tool listed in Section 2.1; additional documents were suggested and shared by steering committee members. From the guidance documents (*e.g*. handbooks and user guides), information abstracted included the purpose, objectives, indicators/metrics measured, estimated time investment, and cost to implement. From the evidence documents, information desired included the year and country/countries of implementation, key findings, successes and challenges with implementation, and any evidence related to the use of the findings and/or recommendations.

Documents targeted for country case studies included, but were not limited to, TB data tool activity reports, presentations, publications, TB program review reports, national TB strategic plans, and Global Fund applications. Country documents were obtained from NTP websites and NTP staff in the case study countries, as well as published English-language journals. Information extracted from reports, presentations, and publications included implementation year, lessons learned, findings, and recommendations from the TB data tools. Information extracted from national strategic plans and Global Fund applications included evidence regarding the use of the TB data tools' findings and recommendations for program planning and monitoring, as well as evidence of planning for upcoming implementation of TB data tools.

#### 2.3.2 Indicator mapping and streamlining

The objectives and indicators/metrics abstracted from guidance documents during the desk review were used for the indicator mapping and streamlining.

#### 2.3.3 Key informant interviews (KIIs)

At the global level, structured KIIs were conducted with experts from major TB technical partners and funders. Key informants were purposely selected following a discussion with the project steering committee to identify key informants that have planned, implemented, and/or analyzed data from TB data tools.

In the country case studies, the project's primary contact at the NTP was asked to identify participants based on outlined criteria. Desired participants included TB program staff who worked at national and subnational levels and TB partners with experience implementing the data tools or using the findings and recommendations of the tools.

Both global and country-level KIIs were conducted using video conferencing. A structured interview guide (Supplementary material 1, 2) was used to direct the interviews and shared with participants ahead of time. It covered perceptions of challenges and gaps in routine TB data systems, the usefulness of various tools in addressing these gaps, and opportunities and challenges with planning and implementing TB data tools. Country interview guides were adapted for each country to reflect the tools that they had previously implemented. The interviews were 60–90 min long, and audio recorded with verbal consent from the participant. Interpreters were provided as needed for key informants in Vietnam.

#### 2.3.4 Use case discussions

One 90 to 100-min use case discussion was conducted in each of the five countries with NTP staff and in-country TB partners. A structured interview guide (Supplementary material 3) was developed and shared ahead of time with participants. Interpreters were available for the use case discussion with Vietnam. The discussions were conducted via video conferencing and audio recorded with prior verbal permission received from all participants.

#### 2.3.5 NTP survey

All countries that had previously implemented at least two unique TB data tools, according to records from partner organizations, were eligible to participate in the NTP survey. Fifty-five countries met this criterion, the majority (83.6%) of which were in Africa or Asia. The NTP managers from all eligible countries were invited via email to participate in the online survey. The email participation request was sent out to non-responsive countries a maximum of four times over 4 months.

The 20-question survey (Supplementary material 4) was designed, conducted, and managed using REDCap electronic data capture tools and was available in English and French, as the majority of eligible countries had one of these as an official language (25, 26). One of the initial questions in the survey provided a checklist of all TB data tools of interest and asked countries to indicate which tools they had previously implemented. Many of the remaining questions were tailored to focus on the country's experience with those specific tools that they had implemented. The survey was estimated to take 20–30 min to complete.

### 2.4 Data analysis and triangulation

Data collection and analysis were conducted concurrently throughout the three phases to explore general themes and tool-based themes within the data.

#### 2.4.1 Desk reviews

The information extracted from the documents was used to summarize and describe the use and impact of the data, findings, and recommendations of the TB data tools and identify data tools whose findings were most useful or impacted program planning and policies. Desk review findings were used primarily to provide context and to triangulate with other findings.

#### 2.4.2 Indicator mapping and streamlining

Key objectives, indicators, and metrics collected by each TB data tool were mapped along the TB care cascade or grouped into the areas of TB burden estimation, program planning, and patient costs. Similarities and any overlap of indicators and metrics across data tools were explored for opportunities to streamline or align across tools. Tool methodology (e.g. facility-based surveys, use of geospatial mapping) across data tools was also explored for opportunities to make tool implementation more efficient.

#### 2.4.3 Key informant interviews and use case discussions

Audio-recorded interviews and use case discussions were transcribed verbatim using NVivo Transcription and re-read and edited for accuracy. The audio recordings were deleted after transcription was completed and reviewed. Inductive content analysis was conducted, starting with systematic coding of interview transcripts by two project staff using NVivo 13 (2020, R1; QSR International, Doncaster, Australia). All initial categories and sub-categories were reviewed, revised, and agreed upon by four project team members (27). The coded interview responses and categories were then systematically explored for commonalities within the data, and key emerging themes (defined as where at least 25 percent of respondents discussed a topic) were summarized (28). The global transcripts and each country's transcripts were coded and analyzed separately and then compared. Two project staff summarized use case discussion responses for each country using discussion transcripts and notes, then compared findings across the five countries.

#### 2.4.4 NTP survey

Survey responses were summarized descriptively with frequencies and proportions. “Other” responses were post coded and open responses were summarized. After reviewing the survey results, project staff had concerns that some respondents may have mistaken the names of some tools for a different tool than the one of interest; and thus, responded to questions on tools that they had not actually implemented. Project staff attempted to verify whether countries had previously completed specific tools by checking with partners that fund or support the tools and then post coding responses accordingly. Only four tools were not able to be verified, including the OneHealth tool for TB budgeting, TB care cascade analysis, epidemiological modeling, and screen-TB. An updated dataset was created using the post-coded responses for the verified tools and the original responses for the four tools that could not be verified. Findings presented here used the updated dataset. Quantitative analyses were conducted using SAS software v9.4 (SAS Institue Inc., Cary, NC, USA).

#### 2.4.5 Triangulation of findings

Findings from country case studies were summarized and compared across countries and then triangulated with the findings from the NTP survey to compose a summary country perspective. Global perspectives consisted of triangulated findings from global KIIs and the global desk review. The global perspective was then compared to the countries' perspective.

The triangulation of input from all data sources was also used to explore potential opportunities for streamlining TB data tools from several perspectives, including but not limited to the tools' objectives, methodology, and target population. Considerations for streamlining included exploring opportunities to combine tools, implement tools with similar sampling strategies at the same time, implement tools that are complementary, and consider logical sequencing of tools to minimize duplicated effort, optimize information gained, and consolidate funding.

A secondary analysis of information collected regarding each of the 17 data tools was conducted using all the data sources, where findings were summarized for each tool.

### 2.5 Development of best practices

A set of practical best practices were developed to guide future planning and implementation of TB data tools. Some best practices were formulated by the project team based on findings and discussions and others were proposed by global and/or country key informants. Draft best practices were shared with steering committee members and their feedback was incorporated.

### 2.6 Ethical considerations

All key informant and use case discussion participants provided verbal consent prior to the interview or discussion. Interpreters were provided as needed for participants in Vietnam. Participants were free to withdraw from the interview or discussion at any time. All interview documentation was anonymized using a unique participant identification number. For each of the case study countries, a letter of support for participation was obtained from the MOH. This activity was reviewed by CDC, deemed not research, and was conducted consistent with applicable federal law and CDC policy (See e.g., 45 C.F.R. part 46, 21 C.F.R. part 56; 42 U.S.C. §241(d); 5 U.S.C. §552a; 44 U.S.C. §3501 et seq.).

## 3 Results

### 3.1 Documents identified for desk reviews

Thirty-two guidance documents for the TB data tools and 67 documents describing experiences with implementing the tools were identified and reviewed during the global-level review. A total of 116 documents from the five case study countries were identified and reviewed. Information that was more frequently available in the documents included the purpose, objectives, implementation period, key data points, key findings, and recommendations of the TB data tools. There was often limited or no information on successes and challenges with implementation, lessons learned, and evidence related to the use of the findings and/or recommendations resulting from the TB data tools.

### 3.2 Respondents in key informant interviews, use case discussions, and NTP survey

Twenty-four global key informants were interviewed. Forty-eight country key informants were interviewed (9–11 per country) and 27 respondents participated in country use case discussions (4–6 per country); there was some overlap in respondents between the country level interviews and use case discussions. The NTP survey was completed by 42 countries (response rate = 76%). Respondent characteristics are described in Table 1.

**Table 1:** Respondent characteristics by data source—TB Data Optimization Project, 2021−2023.

**Characteristics**	**Phase 1: global interview respondents (*n* = 24)**	Phase 2: country case study interview respondents^*^(*n* = 48)	Phase 2: country case study use case respondents^*^(*n* = 27)	Phase 3: NTP survey respondents (*n* = 42)^*^
Duration working in TB (years) Median (IQR)	15 (12–20)	11 (8–15)	n/a	14 (10–20)
**Sex**				
Male	11 (45.8%)	32 (66.7%)	18 (66.7%)	n/a
Female	13 (54.2%)	16 (33.3%)	9 (33.3%)	n/a
**Affiliation (countries)**				
MOH/TB program staff	n/a	31 (64.6%)	18 (66.7%)	42 (100%)
TB program partner	24 (100%)	17 (35.4%)	9 (33.3%)	n/a

* Case study interview participants, use case respondents, and NTP survey respondents are not mutually exclusive.

IQR, interquartile range; MOH, ministry of health; NTP, national TB program.

The median duration of working in TB across all respondents (114) was 13 years (IQR: 9–18). The male to female ratio of global respondents was almost equal, while approximately two-thirds of country case study respondents were male. One third of country case study respondents were TB program partners and two-thirds were TB program staff at the national and subnational level.

Among the five case study countries, three were in Africa and two were in Asia; two countries were low-income and three countries were lower middle-income (29). Among the 42 countries responding to the NTP survey (Supplementary material 5), 22 (52.4%) were from Africa, 13 (30.9%) from Asia, three (7.1%) from Oceania, two (4.8%) from Europe, and two (4.8%) from South America. Ten (23.8%) were from low-income countries, 22 (52.4%) were from lower-middle income countries, and 10 (23.8%) were from upper-middle income countries. Fewer countries in Oceania, Europe and the Americas were eligible to participate in the survey than in Africa and Asia, which contributed to a lower number of respondents from these regions. All NTP survey respondents worked at their country's national TB program but held a range of positions including NTP managers, M&E staff, research staff and other roles.

### 3.3 Respondent familiarity with TB data tools

**Figure 3:**
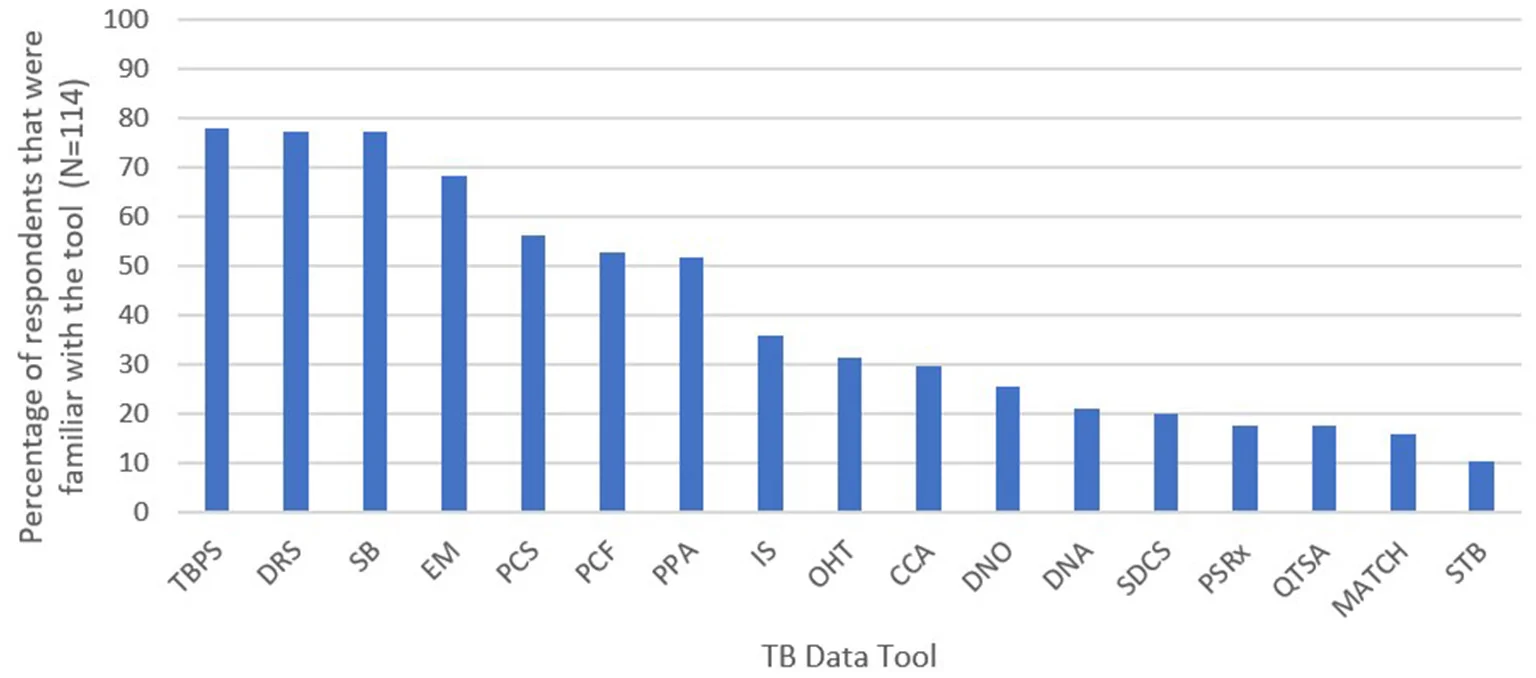
Global and country key informants and survey respondents' familiarity with the 17 TB data tools—TB Data Optimization Project, 2021–2023. TBPS, TB prevalence survey; DRS, anti-TB drug resistance survey; SB, epidemiological review including standards and benchmarks; EM, epidemiological modeling; PCS, TB patient cost survey; PCF, people-centered framework; PPA, patient pathway analysis; IS, inventory study; OHT, OneHealth tool for TB budgeting; CCA, TB care cascade analysis; DNO, diagnostic network optimization; DNA, TB diagnostic network assessment; SDCS, service delivery costing study (Value TB); PSRx, private sector drug sales analysis; QTSA, quality of TB services assessment; MATCH, mapping and analysis of tailored disease control and health system strengthening; STB, screen-TB. Respondents were considered familiar with a tool if they were involved with planning and/or implementing the tool and/or had seen or heard the results of the tool.

The respondents' familiarity with the 17 TB data tools of interest is shown in Figure 3. The most well-known tools were the TB prevalence survey (78.1%), anti-TB drug resistance survey (77.2%), TB epidemiological review (77.2%), epidemiological modeling (68.4%), TB patient cost survey (56.1%), people-centered framework (52.6%), and patient pathway analysis (51.8%). The least well-known tools were the private sector drug sales analysis (17.5%), quality of TB services assessment (17.5%), MATCH (15.8%), and screen-TB (10.5%).

### 3.4 Key themes

Six key themes emerged from the triangulation of findings from the NTP survey, five country case studies, and global perspectives.

#### 3.4.1 Theme 1: usefulness of TB data tools

Although the tools require additional resources such as time, effort, and funding, respondents believed that many tools are worth the investment, because the findings provide countries with critical complementary information. However, one critical finding was that results and recommendations are not consistently used or maximized to their full potential to inform TB program planning and decision making. This was due to reasons such as findings from the tools were not actionable or difficult to address, findings were not followed-up or disseminated to the right audience to make programmatic and policy changes, and/or a lack of funding to implement recommendations. More detailed findings from each of the three data sources are presented in Table 2.

**Table 2:** Respondent perspectives on the usefulness of TB data tools by data source—TB Data Optimization Project, 2021–2023.

**Theme**	**NTP survey findings**	**Country case study findings**	**Global findings**
Usefulness of TB data tools	- Ten of the 17 TB data tools (TBPS, DRS, EM, PCS, PPA, CCA, DNA, DNO, QTSA^*^, SDCS^*^) were considered worth the investment by a majority (≥80%) of countries that have implemented those tools - Respondents were asked about six areas in which TB data tools may be used: (1) understanding gaps in the TB care cascade; (2) routine programmatic planning and forecasting: (3) monitoring progress toward TB targets; (4) developing the TB National Strategic Plan; (5) developing funding applications; and (6) impacting a country's guidelines or policies. Four of the tools (TBPS, DRS, CCA, DNO) were considered very or somewhat useful or helpful across all six areas, but most of the tools scored highly in four of the six areas	- Tools were used to help estimate TB burden, better understand gaps in the TB care cascade, develop the national strategic plan, and write funding applications; but countries also relied extensively on their routine TB data for this - Tools that were cited most often as being particularly useful were: TBPS, DRS, PCS, and PPA; however, these were also the most well-known amongst respondents. Other tools that were cited as particularly useful include: DNO, DNA, IS, SB, and EM - Evidence from desk reviews was somewhat less consistent; not all tools were used/cited in national strategic plans, funding applications, and program reviews	- The most well-known tools were believed by most respondents to be worth the investment (with some caveats, e.g. depending on data availability and a country's needs): TBPS, PCS, CCA, PPA, and SB. Other tools mentioned by many/some respondents as worth the investment were: DRS, IS, PSRx, SDCS, PCF, DNO, and EM - Every tool had respondents who believed it was worth the investment, except for screen TB. However, screen TB was not well-known amongst respondents - Several respondents indicated challenges with operationalizing the findings and optimally implementing recommendations (e.g. findings not actionable or difficult to address, findings not disseminated to the right audience to make programmatic and policy changes, lack of funding to implement recommendations and lack of follow-up after tool implementation)

CCA, TB care cascade analysis; DNA, TB diagnostic network assessment; DNO, diagnostic network optimization; DRS, anti-TB drug resistance survey; EM, epidemiological modeling; IS, inventory study; PCF, people-centered framework; PCS, TB patient cost survey; PPA, patient pathway analysis; PSRx, private sector drug sales analysis; QTSA, quality of TB services assessment; SB, epidemiological review including standards and benchmarks; SDCS, service delivery costing study (Value TB); TBPS, TB prevalence survey.

* TB data tools with small number of NTP survey countries that have implemented the tool (*N* ≤ 5).

#### 3.4.2 Theme 2: opportunities and challenges with planning and implementing TB data tools

Overall, collaboration and alignment amongst stakeholders, receipt of technical and financial support from partners, and capacity building for tool implementation, analysis, and translating findings into action were the most frequently reported opportunities that arose from implementing TB data tools. A country key informant highlighted the benefits of capacity building as follows:

“*If the country could benefit from capacity building during implementation, we don't have to depend on external experts to come…Diagnostic network optimization, epidemiological modelling, and MATCH analysis, those ones would be very important to build capacity so we can routinely implement these activities.”*—Country Key Informant

Conversely, inadequate human resource capacity (technical capacity and number of staff needed) and inadequate financial resources were the most frequently reported challenges. These challenges are illustrated by a country key informant as:

“*The national TB control program has a limited number of technical people on their team…these activities are very technical and require somebody to take ownership and follow up. Although [there are] consultants to conduct these activities, somebody with sufficient knowledge and technical expertise should be there to follow up with the consultants [and] to make sure that recommendations are being implemented and incorporated in national and provincial strategic planning. If there is more technical human resource available, I believe that it will be more helpful and they will be able to distribute the workload. In the last epi review or program review, I have seen that there are only one or two people who are coordinating with the provinces, with the consultants, making sure timelines are being met.”*—Country Key Informant

More detailed findings on opportunities and challenges from each of the three data sources are presented in Table 3.

**Table 3:** Respondent perspectives on significant opportunities and challenges with planning and implementing TB data tools by data source—TB Data Optimization Project, 2021–2023.

**Theme**	**NTP survey findings**	**Country case study findings**	**Global findings**
Significant opportunities from planning and implementing TB data tools	Most significant opportunities (*N* = 42, multiple responses possible) 1. - Technical support from partners: 88.1% 2. - Capacity building for NTP staff: 85.7% 3. - Financial support from partners: 73.8%	- Work with and receive technical and financial support from partners - Capacity building for NTP, subnational level staff, and local partners - Data tools provide additional evidence to inform policy and interventions	- Collaboration and coordination amongst stakeholders (e.g. alignment between partners implementing different tools, MOH working with other government sectors) - Capacity building for country staff
Significant challenges with planning and implementing TB data tools	Most significant challenges (*N* = 42, multiple responses possible) 1. - Insufficient financial resources: 83.3% 2. - Insufficient staff/time: 76.2% 3. - Limited technical capacity: 66.7%	- Limited human resources (e.g. insufficient staff, competing priorities) - Insufficient financial resources (e.g. rely heavily on donor funding) - Do not have required technical capacity to implement tool	- Limited human resources in country (e.g. competing priorities) - Insufficient financial resources for tools (e.g. large surveys are costly) - Limited technical/analytic capacity in country

NTP, national TB program; MOH, Ministry of Health.

#### 3.4.3 Theme 3: technical assistance and financial support for TB data tools

Overall, technical assistance received by countries for TB data tools they have implemented was welcomed and typically reported as sufficient. However, respondents from all three data sources indicated challenges with availability of technical assistance for activities following tool implementation such as completion of reports and implementation of recommendations resulting from the tool.

In terms of financial support, there was agreement across data sources that countries often depend heavily on donor funding for TB data tools due to lack of domestic funding and competing priorities, which can prevent or delay implementation and/or sustainability after initial implementation. Inadequate funding was frequently reported as a significant challenge for planning and implementing TB data tools, as illustrated by country key informants:

“*Unfortunately, funding never seems to be adequate. Sometimes an exercise requires a lot of inputs, it's supposed to take place maybe for an entire year, so we have to do a lot of stretching of the donor.”*—Country Key Informant

“*The biggest problem that we've had is funding, which is largely external. We've not had a lot of in-country commitments from the government…we tend to rely on external funding and have to deal with the changing landscape of funding and sometimes we are not able to get funds in time.”*—Country Key Informant

More detailed findings from each of the three data sources are presented in Table 4.

**Table 4:** Respondent perspectives on technical assistance and financial support for TB data tools by data source—TB Data Optimization Project, 2021–2023.

**Theme**	**NTP survey findings**	**Country case study findings**	**Global findings**
Technical assistance (TA) for TB data tools	- Countries were asked to report whether adequate TA was received *for the tools they had previously implemented*. For many of the tools, 100% of countries that had implemented them reported adequate TA received - The list below shows individual tools with the number of countries that had previously implemented it (*N* =) and the percentage reporting *inadequate* TA received - OneHealth tool (N^*^= 20): 25.0% - Screen-TB (*N* = 5): 20.0% - Epidemiological modeling (*N* = 24): 16.7% - TB patient cost survey (*N* = 21): 9.5% - TB care cascade analysis (*N* = 21): 9.5% - Anti-TB drug resistance survey (*N* = 34): 8.8% - TB epidemiological review (*N* = 39): 2.6%	- Generally received adequate TA to plan and implement data tools - TA is almost always needed and welcomed to implement data tools - Technical support is not always provided for activities after completion of the tool; sometimes, reports aren't completed because consultants leave after tool implementation and analysis	- TA/consultants are almost always externally funded and provide support to countries for implementation of data tools, but not always for analysis and translation of findings into action
Financial support for TB data tools^+^	- Countries were asked to report whether adequate financial support was received *for the tools they had previously implemented*. For many of the tools, 100% of countries that had implemented them reported adequate financial support received - The list below shows individual tools with the number of countries that had previously implemented it (*N* =) and the percentage reporting *inadequate* funding received - TB care cascade analysis (N^*^= 21): 23.8% - Epidemiological modeling (*N* = 24): 16.7% - Anti-TB drug resistance survey (*N* = 34): 11.8% - TB epidemiological review (*N* = 39): 10.3% - OneHealth tool (*N* = 20): 10.0% - People-centered framework (*N* = 10): 10.0% - TB patient cost survey (*N* = 21): 9.5% - TB prevalence survey (*N* = 24): 8.3%	- Lack of domestic funding; heavy reliance on donor funding for data tools - Implementation of data tools are often delayed due to inadequate funding - Donors may not have adequate or earmarked funds for data tools, so countries must make a deliberate effort to get funding for them - Lack of funding to implement recommendations resulting from data tools	- Data tools are almost always donor funded; lack domestic funding for them - Implementation of data tools often delayed due to inadequate funding - Countries can't apply all the data tools they want to due to funding availability - Recommendations resulting from data tools are not optimally implemented due to several issues, including funding constraints

^+^In the NTP survey, countries were asked about tools that they had previously implemented, hence these tools were likely to have been adequately funded since the countries managed to implement them. The data collected did not capture whether there are additional tools they would like to implement but are limited by insufficient funding. Country and global respondents were asked to discuss the need for funding from a more general perspective, and often mentioned challenges with adequate funding to implement recommendations resulting from the tools.

#### 3.4.4 Theme 4: timing and coordination of TB data tools

While there were no questions directly asked in the NTP survey related to timing and coordination of TB data tools, this theme emerged from both case study countries and global respondents. Overall, there was agreement that timing and coordination of TB data tools are challenging and critical. Global respondents highlighted that if multiple tools are implemented around the same time, it is important to ensure good coordination between partners and between partners and the NTP. Respondents emphasized the importance of tool implementation aligning with the country's needs, priorities, and TB strategic planning cycle, as illustrated below:

“*The unfortunate thing with funding, especially from international donors, is sometimes the funding doesn't align with the country's priorities. So maybe in this strategic period, this is what the donor's priorities are, but they do not match the NTP's priorities that year. But then at the end of the strategic period, it's clear that all these supplemental tools should have taken place. So, you end up with multiple tools happening at the same time. Alignment is extremely important.”*—Country Key Informant

“*Over time, the burden of [supplemental tools] is becoming reduced because we have more experience, and we can streamline things…and my thought would be to really think about what the country context is, because you don't want things like epi reviews or drug resistance surveys to become a check the box thing. You want to make sure that they're timed correctly and tailored correctly so that they really meet country needs.”*—Global Key Informant

More detailed findings from each of the three data sources are presented in Table 5.

**Table 5:** Respondent perspectives on timing and coordination of TB data tools by data source—TB Data Optimization Project, 2021–2023.

**Theme**	**NTP survey findings**	**Country case study findings**	**Global findings**
Timing and coordination of TB data tools	This topic was not directly asked in the survey, but 11.9% of the 42 survey respondents reported lack of coordination between partners for tool implementation as a significant challenge with planning and implementing data tools	- Timing of tool implementation is challenging and should align with a country's strategic planning period/Global Fund application cycle; results should be available before the next round of strategic plan development begins so they can inform program planning - Tool implementation should align with the country's TB program priorities; it's important to coordinate with the NTP to ensure buy-in	- Timing of tool implementation is challenging and often not aligned with a country's strategic planning cycle (e.g. tools often take a long time to plan, implement, and analyze results) - When multiple tools are implemented, ensure good coordination e.g. can coordinate analytics/data requests from the NTP

NTP, national TB program.

#### 3.4.5 Theme 5: proposing, deciding, and motivating factors to implement TB data tools

There were mixed opinions from country and global respondents on who typically proposes implementation of TB data tools. The majority of country respondents believed that the NTP most often proposes a TB data tool, while global respondents indicated that suggestions to implement tools typically come from external partners. However, there was strong agreement amongst country and global respondents that the NTP ultimately makes the final decision; while other factors such as funding availability or discussions with internal and/or external TB partners may guide that decision.

When looking at motivating factors to implement TB data tools, the country's need for data or information, suggestions from technical or funding partners, and the availability of funding to implement the TB data tool were the most frequently reported motivating factors. A country key informant highlighted the importance of having information for decision making:

“*The most motivating factor at the program level has been to strengthen our evidence base. We want to ensure that we have as much information as possible so that we can enhance our position for decision making. We understand and sometimes have suffered where inaccurate policies can prove to be costly, not just financially, but also in time.”*—Country Key Informant

More detailed findings from each of the three data sources are presented in Table 6.

**Table 6:** Respondent perspectives on proposing, deciding, and motivating factors to implement TB data tools—TB Data Optimization Project, 2021–2023.

**Theme**	**NTP survey findings**	**Country case study findings**	**Global findings**
Who typically proposes implementation of TB data tools and who makes the final decision to implement TB data tools	- Most countries reported that the NTP/MOH initially suggests (76.2%) and makes the final decision (90.5%) to implement a TB data tool - Six out of 42 countries (14.3%) reported that international technical partners and in-country technical working groups most often suggest tools, but do not make the final decision. Three out of 42 countries (7.1%) reported that funding partners have the final decision to implement a tool	*Who typically proposes* - The NTP typically proposes implementation of a tool based on a need for information - External technical partners sometimes have an idea and propose it to the NTP - The TB technical working group in the country usually gives advice and recommendations to implement a tool *Who makes the final decision* - NTP/MOH leadership makes the decision/gives approval to implement a tool, but it is usually discussed first with in-country partners or a technical working group; the approval process in some countries is challenging - Sometimes the NTP/MOH is advised by external or internal TB partners or a technical working group - Implementation depends on whether donors support the idea and provide funding	*Who typically proposes* - Tools are usually recommended by technical partners or are a requirement from funders, which often leads countries to implement tools *Who makes the final decision* - Initial push comes from external partners but the ultimate decision rests with the NTP. - The NTP should be making the decision, but this is not always the case; NTPs have become more engaged over time as they have seen tools being implemented in other countries, so there is more internal motivation now
Motivating factors to implement TB data tools	Strongest motivating factor (*N* = 41^*^) 1. - Need for data/evidence: 51.2% 2. - Measure progress toward global TB strategies/targets: 34.1% 3. - Availability of funding: 12.2% 4. - Request by donor: 2.4%	- The country's desire for more information for planning, decision making, policy making, designing interventions, or understanding the TB situation - External push/ recommendation from technical partners or requirement for funding application - NTP or leadership's focus or interest - Ability to evaluate progress toward global TB strategies (e.g. END TB) - Availability of funding to implement the tool; dependent on donor support for tools	- External push from technical and/or funding partners - Funding to implement the tool - Internal motivation within the NTP (e.g. data needs for upcoming national strategic plan development); if a country has strong NTP leadership and pushes for program needs

NTP, national TB program; MOH, Ministry of Health

* Excluded one “other” response as the response entered was unclear.

#### 3.4.6 Theme 6: routine data systems and their relationship to supplemental data tools

Overall, country and global respondents believed that top challenges/gaps in routine TB data systems include limitations in data utilization (e.g. limited analytic capacity in staff, data quality) and limitations in the data systems themselves (e.g. fragmented data systems, paper-based recording and reporting, timeliness of data, limited variables collected). Country and global respondents want to invest in strengthening routine data systems which are more sustainable, enable the availability of robust and timely electronic case-based data, and can support TB program planning. However, even with stronger routine systems that may decrease the need for some data tools, other tools will still be needed to provide supplementary data. The importance of strengthening routine data systems is illustrated by country and global key informants:

“*I think supplemental tools are important, but there are lots of recommendations in these tools that if we don't strengthen the routine systems, we will not move…if we don't strengthen information or social behavior change activities in the communities, we will do another prevalence survey and find the same information.”*—Country Key Informant

“*The priority now is to have a good surveillance system which also facilitates analysis and interpretation of the data. It means a surveillance system with an automated fiscal dashboard that will allow people with minimal capacity of doing analysis to be able to use the results.”*—Global Key Informant

There was no consensus over what information is still missing or not captured by routine data and supplemental tools, but needed for TB program planning, as it depended on each country's needs. Several case study and NTP survey respondents reported the need for subnational level estimates for better target setting and program planning. Many global respondents believed there are already too many TB data tools and that there is no need for developing new tools; however, this sentiment did not emerge as strongly from country respondents. More detailed findings from each of the three data sources are presented in Table 7.

**Table 7:** Respondent perspectives on routine data systems and their relationship to supplemental data tools—TB Data Optimization Project, 2021–2023.

**Theme**	**NTP survey findings**	**Country case studies**	**Global perspective**
Challenges/gaps in routine TB data systems	Top 5 challenges (*N* = 42, multiple responses possible) 1. - No real-time data: 66.7% 2. - Data quality: 64.3% 3.- Limited analytic capacity at lower levels: 52.4% 4. - Limited use of data at lower levels: 52.4% 5. - Use of paper-based system: 45.2%	- Limited variables collected by systems (e.g. do not collect socioeconomic status, comorbidities, patient costs, quality of care) - Fragmented data systems (e.g. TB data not integrated with other diseases, not linked with lab data) - Aggregate, rather than case-based reporting - Mode of collection: Some countries still using paper-based systems or transitioning to digital systems - Data quality (e.g. duplicate data, lab and clinical data don't match)	- Fragmented data systems - Some countries still using paper-based systems - Limited analytic skills of TB program staff at national and lower levels; also impacts data use - Data timeliness (e.g. reporting timeline inadequate for decision making, no real-time data) - Data flow (e.g. data only flows bottom-up)
Strengthening routine TB data systems vs. implementing TB data tools	Respondents were asked: If they received a sum of money for TB data activities, what percentage should be allocated toward strengthening routine data systems vs. implementing data tools? (percentage of respondents selecting each range shown below) (*N* = 42) [n] - Allocate >75% toward routine systems: 21.4% - Allocate 51%−75% toward routine systems: 40.5% - Allocate 26%−50% toward routine systems: 28.6% - Allocate < 25% toward routine systems: 9.5%	- Ideal to strengthen routine systems because they are more sustainable than data tools; more robust systems mean certain data tools will no longer be needed - Need to ensure systems are integrated and user-friendly, not just strengthened - Important to find a balance; some data tools will be needed even if routine systems are strengthened - Donors and the government should invest in both strengthening routine systems and in implementing critical data tools	- Ideal to strengthen routine data systems, as it eliminates the need for some data tools; strengthened systems allow for better or more analyses - Invest in routine data systems to have high quality data for the long run, but in the meantime, data tools are still needed for decision making
Information still missing from routine data and TB data tools and/or needed for TB program planning	- Missing/needed information (*N* = 42, question was open-ended) - Subnational level estimates (*n* = 4) - Private sector data (*n* = 3) - Data on presumptive TB (*n* = 3) - True TB burden (*n* = 2) - Case-based data (*n* = 2) - Hotspot mapping (*n* = 2) - Cost benefit/effectiveness (*n* = 2) - Nothing (*n* = 2) - Other responses received from individual countries: stigma measurement, quality of care, impact and outcome information, implementation effectiveness, true number of missed/undiagnosed cases, close contact follow-up, geospatial mapping, social determinants, comorbidities, health seeking behavior, patients lost to follow-up	- Subnational level estimates - TB-related mortality - Additional variables for data system such as patient socioeconomic status and comorbidities - Integration of private sector data into national TB surveillance data	- No need for new tools; more important to optimally use existing data for decision making and to provide guidance/training to countries to do so - Quality improvement/quality of care^*^ - Stigma Assessment^*^ ^*^Tools to measure this already exist

### 3.5 Additional analyses

#### 3.5.1 Findings by TB data tools

Overall, feedback on most of the TB data tools was positive regarding the tools' impact and usefulness. Only a few of the tools had mixed findings, and none of the tools had predominantly negative findings. Feedback was limited for a few of the TB data tools that were less well-known by respondents and they were therefore unable to comment on these tools (e.g. MATCH, screen TB). More detailed findings from this secondary analysis are presented elsewhere (30).

#### 3.5.2 Opportunities for streamlining of TB data tools

A total of 164 key objectives and metrics were abstracted from the tools' guidance documents during the desk review and mapped to steps of the care cascade and three additional categories- burden estimation, program planning, and patient costs. Findings from this mapping showed limited overlap between tools. Even where indicators were similar, the underlying data sources or methodology were different.

Global and country key informants were asked if any of the TB data tools could be combined. The majority of respondents agreed that although it would be ideal to combine tools to save resources (e.g. funding, time, effort), it would be a difficult endeavor as each tool has its own purpose and uses a different methodology. Nevertheless, examples of suggested combinations included TB prevalence survey and anti-TB drug resistance survey, TB prevalence survey and TB patient cost survey, TB diagnostic network assessment, diagnostic network optimization, and MATCH, and TB patient cost survey and TB service delivery costing study.

However, with consideration of each tool's methodology, objectives, and metrics, many of these combinations are not feasible. For example, TB prevalence surveys and anti-TB drug resistance surveys are both nationwide surveys but have different target populations. TB prevalence surveys target the general population and have a much larger sample size, while anti-TB drug resistance surveys target new and re-treated TB patients at the time of diagnosis. Anti-TB drug resistance surveys also need to target a larger number of persons with TB than captured in a TB prevalence survey. Similar limitations apply to the other suggested combinations.

Although one tool cannot simply replace another, opportunities for making implementation more efficient were identified. Some ideas are to implement tools with similar sampling strategies at the same time, implement complementary tools in a collaborative manner to minimize data requests and maximize information gained, and to consider logical sequencing of tool implementation (e.g. implement those that generate primary data first, so they can feed into secondary data analysis tools). Potential opportunities and considerations for streamlining TB data tools are presented in Table 8.

**Table 8:** Opportunities for streamlining of TB data tools—TB Data Optimization Project, 2021–2023.

**Opportunities for streamlining/implementing in a complementary manner**	**TB data tools**	**Feasibility/considerations**
**Facility-based surveys and assessments** - Could conduct surveys/assessments at the same time to use the same logistic support (e.g. data collectors, travel costs, patients sampled) and methodology (e.g. patient/staff interviews).	TB patient cost survey (PCS) - Quality of TB services assessment (QTSA) - Anti-TB drug resistance survey (DRS)	- Implementation will be more complex if administering more than 1 survey/assessment - Need to ensure length of patient interviews does not become burdensome (consider abridged versions of surveys/interviews) - Patients sampled for PCS include both those on intensive and continuation phase; DRS includes patients at time of diagnosis; QTSA does not specify a specific time point during treatment for interviews
**TB data tools that use geographical mapping** - Potential overlap in inputs could be considered to maximize efficiencies of data collection and minimize data requests	- TB diagnostic network assessment (DNA) - TB diagnostic network optimization (DNO) - MATCH	- The DNA and DNO have different aims for their mapping exercises, and the intended scopes can be assessed to ensure complementarity - Output from a DNA can often feed into a DNO
**TB data tools that use existing data to assess potential gaps in the TB patient pathway** - Complementarity of tools could be integrated to minimize data requests and maximize output	- MATCH - Patient pathway analysis (PPA)	- MATCH applies mapping and spatial analysis techniques to existing health data to identify gaps and then target interventions. Core to this approach is the integration of multiple sources of sub-nationally disaggregated data to identify people with TB who are missed along the TB patient pathway. The PPA also uses existing data to assess how well-aligned available diagnostics and treatment services are with where patients present for care, hence these tools could be integrated to provide a fuller picture of the gaps - Implementers would need to have expertise in both analyses

### 3.6 Key take home findings

After thematic findings were triangulated from all data sources, they were distilled into eight key take home findings (Box 1).

Box 1 **Key take home findings from the TB Data Optimization Project, 2021–2023.**

- TB data tools are critical to fill gaps, are complementary to routine TB data, and require substantial resources (time, funding).
- Supplemental TB data tools have limited overlap; one tool cannot simply replace another tool, though tools can be implemented in a complementary manner.
- Countries need more and better quality subnational level data for better target setting and program planning.
- Lack of resources and feasibility constraints result in limited implementation of recommendations from TB data tools.
- Additional capacity building is needed in countries to implement supplemental TB data tools, including planning, implementation, analysis, data interpretation, dissemination, and translation of findings into action.
- Funding for TB data tools is largely from donors; they may not always be funded at the right time due to funding availability and/or interest.
- Improved coordination and better aligned timing of different TB data tools are necessary to optimize use of their findings for National Strategic Plan development, funding applications, and program planning.
- Strengthening routine data systems (preferably digital and case-based), is fundamental for attaining robust, sustainable TB program data.

## 4 Discussion

To our knowledge, this is the first time such a project has been implemented to better understand the overall use and practicality of a spectrum of TB data tools. NTPs and TB partners recognized the utility and value of TB data tools, including their usefulness for burden estimation, understanding and addressing gaps in the TB care cascade, and program planning. Although TB data tools help to fill gaps in routine data systems, there was consensus that routine data systems could be strengthened and integrated to facilitate better data collection and analytic capacity (e.g. transition from paper-based systems to case-based digital systems, improve data quality, real-time reporting, data dashboards to assist with data analytics and interpretation, and integrate TB data systems with other disease and laboratory data systems). Furthermore, having high quality subnational routine data could improve target setting and program planning for countries. Strengthened routine data systems will result in some tools becoming redundant; however, other tools will still be needed as they answer questions that cannot be assessed with routine data systems. Additionally, some countries still do not have or are transitioning to a high coverage and quality digital case-based system and their focus is on strengthening routine systems; however, they can still implement different TB data tools to address specific needs in the interim.

Despite the considerable efforts and resources needed to implement TB data tools, the overall response to these tools was very positive. However, results and recommendations from the tools are often not used to their full extent and there is room for improvement in alignment of timing, coordination, and prioritization of TB data tools so that results can be used for national strategic plan development, program planning, and funding applications. Data tools are highly dependent on partner funding and technical assistance which have been successfully mobilized for most tools that countries have implemented but can be a limitation in sustainable implementation of these tools.

Building local technical capacity and empowering NTP ownership are important for both implementing these tools and also using the results and recommendations to their full extent. NTPs view capacity building as an opportunity, and it would enable the NTP to implement the tools with less reliance on external consultants. Although countries often received sufficient technical assistance for tool implementation, external consultants often do not have funding or time to follow-up on completion of reports and translation of results and recommendations into action. It is therefore important to extend technical support to implement recommendations. In addition, although sufficient funding was typically received for tools that countries have implemented, some countries experienced delays due to timing of the funding or inadequate funding received initially. There was often inadequate funding to implement recommendations resulting from the tools. There is heavy reliance on partner funding due to limited domestic funding, competing priorities, and commitment from the government for implementation of TB data tools.

Prior to the design and implementation of this project, substantial overlap between TB data tools was anticipated. However, findings showed limited overlap and no simple way to combine different TB data tools. Even if tools have some overlap in objectives and/or metrics, primary objectives and methodology differ. However, there are opportunities to save resources (e.g. costs, person-time) and streamline efforts by simultaneously implementing tools with similar target populations. Additionally, coordinating the implementation of complementary tools can minimize data requests and maximize information gained. For example, a combined MATCH and patient pathway analysis in Madagascar provided a mixed methods assessment of healthcare-seeking behavior, the availability and coverage of TB diagnostic and treatment services as well as identification of geographical areas where people with TB are likely to remain undetected along the care cascade. Findings from the combined analyses were used to inform regional and national TB service delivery in efforts to improve screening, diagnosis, linkage, and treatment outcomes, such as the installation of additional GeneXpert machines in identified high-priority regions (31). In Uganda, a TB diagnostic network assessment and quality of TB services assessment were both implemented in 2019; later, an analysis was completed to understand how the two assessments could be used synergistically by comparing the objectives, content, and results. Findings from the analysis revealed that the performance indicators from the two assessments do not completely align; however, it was still useful to compare the findings from both to evaluate the performance and quality of the country's TB diagnostic services (32). Also, in Cambodia, a patient pathway analysis and care cascade analyses for both TB disease and TB preventive treatment (TPT) were completed to identify gaps in care-seeking, coverage, and access to TB services and TPT, which informed action plans to improve the TB response in the country. Both analyses used existing data from the TB prevalence survey, routine surveillance and program data, the global TB database, and published articles (33, 34).

### 4.1 Strengths and limitations

This project had several strengths. It is the first effort to systematically review and document the use and usefulness of collectively available TB data tools. It included perspectives of global and country respondents from different geographical regions, used both quantitative and qualitative methods, and findings from all data sources were triangulated to better understand the use and usefulness of TB data tools (35, 36).

This project also had several limitations. First, the project was designed to look at the overall process of tool planning, implementation, and use of findings and recommendations; therefore, many tools were covered, and as a result, information on each individual tool is limited. Next, the project was designed in 2020 and early 2021, and that is when selection of the focus tools took place. The focus tools were selected based on their prior implementation by multiple countries and recommendations by partners active in the TB data realm, as it was not possible to include all potential sources of TB data. To our best knowledge, there were no commonly used tools that focused on TB prevention at that time, and therefore none were included in the project. Furthermore, additional tools have been developed since then that were not included in the project [e.g.Clinic-Lab Interface Continuous Quality Improvement (CLICQ!/Diagnostic Cascade Evaluation (DiCE) Toolkit] (37, 38). Another limitation was concern that some NTP survey respondents reported experience with a tool that they had not actually implemented, due to some tools having similar or non-specific names. To mitigate this, completion of a tool was verified by the tool's developer/implementer where possible, and answers to tool-specific questions were limited to countries with verified completion of the tool. Some TB data tools were also less known among respondents and thus discussed very little in interviews (e.g. private sector drug sales analysis, quality of TB services assessment, MATCH, screen TB). Hence in questions about the “most useful” tools, it was difficult to distinguish whether these tools were considered less useful or were just less well-known or had less funding behind them. Comments on these tools were also based on only a few respondents. Lastly, since respondents were asked about tools that had already been implemented, recall bias and staff turnover could have affected respondents' recollections of information and experiences. To mitigate recall bias, tool descriptions and implementation dates were provided when relevant. A few respondents mentioned tools their country was planning to implement, though respondents were not directly asked about upcoming plans. This was a missed opportunity.

## 5 Best practices

### 5.1 Best practices resulting from the findings

Best practices derived from the project were grouped into six areas: general, optimizing the usefulness of findings and recommendations resulting from TB data tools, timing and coordination of tool implementation, capacity building in countries, funding of tools, and conducting tools at subnational level.

#### 5.1.1 General best practices

1. Before considering which TB data tools to prioritize, it is important to review existing data, including routine programmatic data and previously implemented TB data tools and research. It is helpful to map and consolidate all existing data, so that key data and evidence gaps can be identified.
2. Not all data tools need to be implemented in all countries; it is important to carefully prioritize activities based on existing data gaps and country priorities.
3. It is important that the NTP is invested in any tool to be implemented, and fully understands the type of findings and recommendations that it generates.
4. As TB programs and routine data systems are strengthened, some data tools may no longer be needed. Countries that transition from an aggregate or paper-based to a case-based digital surveillance system may be able to collect and analyze data to answer specific questions, which may make certain tools redundant.
5. It may be possible to add aspects from one tool onto another tool to decrease the total number of tools to be implemented. However, a thorough assessment of logistical considerations to implement a combined tool can help determine if this is feasible.
6. If no data tool exists that directly addresses a priority question or data gap, it may be possible to integrate additional questions into an existing tool, or additional variables into routine program data. Alternatively, a research study to address the specific gap could be considered.
7. It may be helpful to consider tools and studies that can be implemented with minimal technical assistance and financial support, so that countries are less dependent on partners to implement them.

#### 5.1.2 Improving the usefulness of findings and recommendations resulting from TB data tools

1. When contemplating whether to implement a data tool, assess whether prior recommendations from that tool and related tools have been implemented. If prior recommendations have not been implemented, repetition of the tool will likely generate the same recommendations rather than new ones.
2. In addition to resources needed to implement a data tool, it is important to consider the resources that will be needed to implement recommendations derived from the tool.
3. It is important to involve technical working groups in the development of recommendations and action plans.
4. It is helpful if recommendations resulting from the tools meet SMART criteria: specific, measurable, actionable/achievable (feasible), relevant, and time bound (39).
5. It is important to designate a responsible party/parties to implement recommendations.
6. It is important to disseminate findings and recommendations and in a timely manner, to all relevant internal and external stakeholders, with requests for support to implement recommendations.
7. It is beneficial to consider recommendations from the data tools in the development of National Strategic Plans and funding applications.
8. It is important to translate relevant findings into digestible key messages for civil society and the general public. Consider requesting funding and technical assistance to interpret and disseminate results with engagement from civil society for advocacy, program implementation, and National Strategic Plans.

#### 5.1.3 Timing and coordination of TB data tools' implementation

1. It is important that in-country partners coordinate with each other and the NTP to ensure they support activities that are a priority for the NTP, and that implementation of multiple data tools does not place undue burden on the NTP.
2. NTP approval for data tools is critical; without it, the resulting recommendations are less likely to be implemented.
3. It is important to identify a logical sequence and timeline for tool implementation, so that findings are available for the next National Strategic Plan development or mid-term review, and results from primary data collection tools can feed into data tools that use secondary data. This timeline may be included in National Strategic Plan and funding applications to ensure a logical, integrated approach.
4. It is helpful if multiple stakeholders coordinate the implementation of data tools or requests in a country to reduce the overall burden, avoid duplication of efforts, and promote cost sharing.

#### 5.1.4 Capacity building in countries for planning, implementation, analysis, and interpretation of data/findings of TB data tools

1. It is important to include staff from national and subnational levels, as well as partners, in the planning and implementation of tools.
2. It is important to ensure that planned technical support for activities continues through analysis, dissemination, report writing, and implementation of recommendations.
3. When providing technical assistance to implement data tools, consider capacity building of local staff to implement the tool, analyze the data, and translate findings into action.
4. Consider South-to-South collaborations with technical support provided by trained/experienced persons from neighboring countries.

#### 5.1.5 Funding of TB data tools

1. It is important to incorporate data activities into national strategic plans and:
  a. Advocate for domestic funding.
  b. Include funding requests in Global Fund and other donor applications to minimize the need for *ad hoc* funding.
2. It is important that partners consider alignment of their funding with the country's needs and priorities, rather than being driven by donors' preferences.

#### 5.1.6 Implementing TB data tools at subnational level

1. Some data tools could be suitable to implement at subnational levels (or to estimate subnational indicators) to better understand issues at subnational levels. However, it may be overly expensive to generate subnational estimates in a methodologically sound way for some tools like TB prevalence surveys and anti-TB drug resistance surveys, although a very limited number of strata might be feasible.
2. There is substantial need for subnational estimates for program planning. For reliable subnational estimates, it is important to ensure that subnational routine data is high quality. In addition, when tools are being developed it is important to consider whether methodologically-sound processes to obtain subnational estimates can be incorporated into the tool.

## 6 Next steps

Findings and best practices from this project have been disseminated and discussed in various fora with countries and partners that support the funding and implementation of TB data tools. In addition, the findings and best practices are being used to inform development of a framework that will guide NTPs in understanding which TB data tools can address identified data gaps, then prioritize and plan for TB data tool implementation. The aim is to make selection and implementation of TB data tools more efficient and informed, as well as build capacity for NTPs to drive this process. Ultimately, the longer-term goal remains the adoption, transition and/or strengthening of the routine case-based digital surveillance systems and improving the quality of the data and its use for programmatic decision-making.

## Data Availability

The datasets presented in this article are not readily available due to the nature of the qualitative and quantitative data. The data will not be made publicly available since the identities of respondents could potentially be guessed from the raw content of an individual's responses. Requests to access the datasets should be directed to rfiorillo@cdcfoundation.org.
