# Supplementary material for: Optimizing use of data and evidence-related tools to complement routine surveillance systems for tuberculosis program planning
Source: Front Tuberc. 2025 Jul 17;3:1622167. doi: 10.3389/ftubr.2025.1622167 (PMC13491421; doi:10.3389/ftubr.2025.1622167)
Supplement: Supplementary file 1 [file Table_1.docx]

# **Supplementary Material 1: Global key informant interview guide**

## Project ID:

## Date:

## Time:

## Key Informant Interview Questions for Global Partners

***Some things to keep in mind during the interview****:*

- *We’re interested in your experiences with TB data collection tools in low and middle-income countries that have used, or would be candidates to use, the tools and activities on the pre-interview questionnaire.*
- *Definition of “useful”: Accurate and timely data that directly informs decision-making because they can be accessed and understood by stakeholders during program planning and decision making.*

*“I will start by asking you about routine TB surveillance systems and then we will focus our discussions on TB supplemental tools and activities. When we say supplemental tools and activities, we are talking about all the surveys and tools that we asked about in the questionnaire you completed, such as TB prevalence surveys and Drug Resistance surveys.”*

**Interview questions:**

1. Thinking about the **routine** TB data collection systems that countries use, what are the biggest challenges, limitations, and gaps in the **availability and** **use** **of data for TB program planning and decision making**?

*“Thank you. We will now shift our focus to the TB supplemental tools and activities.”*

2. Thinking of these challenges, limitations, or gaps, **can you tell us how supplemental tools and activities have been useful in addressing these or other important challenges**?

*[Start open-ended; but probe with tools that respondent doesn’t include in their answer]*

*[Probe about other challenges that tools can address – not limited to challenges listed above]*

3. Thinking about these supplemental tools and activities, can you tell us two things: first, what is typically the motivating or driving factor for countries to implement these supplemental tools/activities? And second, who – as in the NTP and/or other specific partners – are typically involved in deciding to implement this activity in a country?

[Probe: Does this differ by tool/activity? Can you provide specific examples?]

4. Can you describe common challenges and opportunities encountered with planning and implementing these activities? Challenges and opportunities can include those at the country level as well as challenges and opportunities for funders and partners that are supporting the activities.

[If not already mentioned, probe for: funding challenges, implementation challenges, burden on countries]

[Follow-up question if they don’t address specific activities: is there anything related to specific activities that you would like to add?]

5. Do you feel that the programmatic recommendations and decisions resulting from these tools are optimally implemented? If no, why? This is a general question, but feel free to speak about specific tools if you’d like.

[Follow-up question if no: Do you have any suggestions on how this could be improved?]

6. **Considering the full spectrum of supplemental activities and the burden they may place on countries, funders, and partners,** what do you think of the overall frequency and timing with which these supplemental activities are typically implemented in countries?

[Prompt if needed: too frequent or not frequent enough]

7. Given that these activities require significant human and financial resources, do you think these activities are generally worth the investment? Please also tell us any key country characteristics that would influence your response. We’d like your answer to be tool-specific, so let’s go down the list of activities that you said you were familiar with.

| *TB Tools and Activities* | *Worth the investment* |
| --- | --- |
|  |  |
|  |  |

8. Do you think that more resources should be invested into strengthening routine systems, even if it means that there may be fewer resources available for supplementary activities like the ones we are discussing today? Please give us some reasons for your answer.
9. Given the burden that these activities can place on countries, do you think there are opportunities for combining or streamlining activities with **related goals or similar methodology** to reduce the overall burden? If yes, can you tell me which tools have potential opportunities to be combined or streamlined?
10. Given all that we have discussed, are there activities that you think should be prioritized for implementation? If your response varies depending on country characteristics, please tell us the country characteristics or other factors that influence your prioritization of activities.

[Prompt with specific tools if needed]

11. Do you think there is a need to develop any new supplemental tools or activities to address gaps in routine programmatic data? If yes, what programmatic areas would benefit from new tools or activities?
12. Finally, I’d like you to consider a **broader scope** of TB data needs – think about existing data systems and how they are used, as well as the role of supplemental activities. From your perspective, what are the top data-related priorities moving forward to ensure that countries have and use “need to know” data for program planning and decision making?

[Open ended response]

13. Is there anything else that you would like to tell us about the use and usefulness of these supplementary tools that you think is important to consider as part of this project?
