# Supplementary material for: Optimizing use of data and evidence-related tools to complement routine surveillance systems for tuberculosis program planning
Source: Front Tuberc. 2025 Jul 17;3:1622167. doi: 10.3389/ftubr.2025.1622167 (PMC13491421; doi:10.3389/ftubr.2025.1622167)
Supplement: Supplementary file 2 [file Table_2.docx]

# **Supplementary Material 2: Country key informant interview guides for national and subnational level**

## Project ID:

## Date:

## Time:

## Key Informant Interview Questions for Persons Working at the National Level

***Some things to keep in mind during the interview****:*

- *We’re interested in your experiences with TB data collection tools and activities in your country.*
- *Many of the questions in this interview will focus on “supplemental” TB data tools and activities. When we refer to “supplemental” activities, we are talking about surveys and data analysis activities that are not part of your routine data collection and analysis systems; these supplemental activities are implemented occasionally to provide supplemental information. Based on the reports we have reviewed, it looks like the recent supplemental TB data activities that have been implemented in [insert country] include [insert list of activities].*

**Do you have any questions before we start?**

**The first few questions are just to help us understand your background in TB and your experience with the supplemental tools that have been implemented in [insert country].**

1. Can you tell me how many years you have worked in positions or on projects that involve TB? ________ (years)
2. What is your current role?
3. Can you tell me how many years you have been in your current role? ________ (years)
4. Next, we will go through a list of supplemental TB data tools and activities that have been implemented in [insert country]. For each of these, could you tell me which of the following 3 categories best describes your experience with the specific activity?

a) Directly involved with planning or implementing the activity.

b) Not directly involved with planning or implementing, but have seen/heard the results or findings. *For key informants from the MOH*: if you approved or endorsed an activity and were informed of the findings, but not involved with implementing the activity, please choose this option.

c) Not involved and do not know what the results/findings were

List of tools or activities recently implemented in country *(to be pre-populated)*:

| **Tool/Activity (year)** | **Role** |
| --- | --- |
|  | ☐ Was involved with planning or implementing  ☐ Was not involved with planning or implementing, but has seen/heard the results or findings  ☐ Not involved and do not know what the results/findings were |
|  | ☐ Was involved with planning or implementing  ☐ Was not involved with planning or implementing, but has seen/heard the results or findings  ☐ Not involved and do not know what the results/findings were |

**Now we will start the open-ended interview questions.**

*“I will start by asking you about routine TB surveillance systems and then we will focus our discussions on TB supplemental activities you are familiar with. When we say supplemental activities, we are talking about all the surveys and tools that we asked about earlier.”*

1. Thinking about the critical information desired for TB program planning and decision making, what information is **not provided by the routine data collection systems in [country]**?

[Prompt if didn’t mention: limitations in ability to use data/Is there anything that makes it hard to use the data?]

*“Thank you. We will now shift our focus to the TB supplemental activities.”*

2. Can you tell us **if and how the supplemental activities** that were conducted in [country] have provided some of this critical information for the country?

3. Thinking about these supplemental activities, can you tell us two things:
   a. First, what is typically the **motivating or driving factor** to implement these supplemental activities? Does this differ by activity?
   b. Second, **who is typically involved in deciding** to implement such activities in your country? Does this differ by activity?
4. Can you describe **common challenges and opportunities** you have encountered with planning and implementing these supplemental activities?

[Follow up (if NTP staff): How does implementing supplemental activities affect the NTP’s workload?]

[Follow up (if partner): What is your impression of how these supplemental activities impact the NTP’s workload?]

5. How have the findings and recommendations from these supplemental activities typically been **disseminated?**

Follow-up: do you think that the following groups of people have been adequately informed of the findings and recommendations:

-NTP staff at national level

-TB program staff at lower levels

-Partners involved in supporting the TB program

6. **Thinking about the different supplemental activities you have implemented in recent years**, what do you think of the frequency and timing with which these supplemental activities were implemented?

Please comment on what you think of:

a. The frequency and timing of each tool
b. The timing related to other tools or planning cycle

[*Probe if they did not mention***:** For which of the tools would you recommend implementing more than once, e.g. to assess change over a specified time period? Are there any would you recommend implementing on a routine basis (e.g. once a year)?]

7. If you were to implement (or not) any of the supplemental activities again, **would you do anything differently and why**? We will go through the list of activities from before.

[*Probe: ask about changes both in terms of implementation as well as use of outcome/results]*

8. Do you think the **recommendations from these supplemental activities have impacted TB program planning and decision making**? If yes, how? If no, why not? Are there any activities that have been particularly important or useful for your country?

[*For those who already participated in use case discussion; re-phrase as*: We discussed the use of these tools for TB program planning and decision making during the group discussion on use case questions, is there anything that you want to add to what was said during the group discussion?]

[*If not mentioned*: ask about tools they are familiar with]

9. Do you think that **more resources should be invested into strengthening routine systems**, even if it means that there may be **fewer resources available for supplementary activities?** Please tell us if your response depends on whether the activity is funded domestically or by donors.

[Probe – if only give short yes/no answer, ask “can you give us some reasons for your answer?”]

10. Do you feel that it was necessary to implement all the different supplemental tools/activities the country has done, **or could some of these activities have been combined because they provided similar information or used similar methods to collect data?**
11. What would you like donors/international stakeholders and other countries to know about your experience with the various supplementary tools/activities?
a. Donors/international stakeholders
b. Other countries
12. Is there anything else that you would like to tell us about the use and usefulness of these supplementary tools that you think is important to consider as part of this project?

## Project ID:

## Date:

## Time:

## Key Informant Interview Questions for Persons Working at the Subnational Level

**The first few questions are just to help us understand a little about your background working in TB.**

1. Can you tell me how many years you have worked in positions or on projects that involve TB? ____________ (years)
2. What is your current role?
3. Can you tell me how many years you have been in your current role? ________ (years)

**Next, we would like to understand your experience with supplemental TB data activities that have been implemented in [*insert country*].**

- ***Supplemental Activities*** *are surveys and data analysis activities that are not part of your routine data collection and analysis systems.*
- *These supplemental activities are implemented occasionally to provide additional information, mostly at the national level.*
- *These supplemental activities are often implemented with assistance from WHO, USAID, Global Fund, as well as local universities and research organizations.*
- *Supplemental activities [insert country] has implemented include: [insert list of activities].*

**Do you have any questions before we start?**

4. Now we will go through a list of supplemental TB data activities that have been implemented in *[insert country]*. For each of the activities we would like to know how familiar you are with this activity.

List of tools or activities recently implemented in *[insert country]* (*to be pre-populated*):

| **Tool/Activity** (year/description/regions included) | **Role** |
| --- | --- |
|  | Were you aware of this activity being implemented in *[insert country]* in *[year]*:  ☐ Yes  ☐ No [skip to next tool] |
|  | Were you aware of this activity being implemented in your region?  ☐ Yes  ☐ No |
|  | Your experience with the specific activity?  ☐ Was involved with planning or implementing  ☐ Was not involved with planning or implementing, but has seen/heard the results or findings  ☐ Not involved and does not know what the results/findings were |
|  | Were you aware of this activity being implemented in *[insert country]* in *[year]*:  ☐ Yes  ☐ No [skip to next tool] |
|  | Were you aware of this activity being implemented in your region?  ☐ Yes  ☐ No |
|  | Your experience with the specific activity?  ☐ Was involved with planning or implementing  ☐ Was not involved with planning or implementing, but has seen/heard the results or findings  ☐ Not involved and does not know what the results/findings were |

**Now we will start the open-ended interview questions.**

*“I will start by asking you about routine TB surveillance systems and then we will focus our discussions on TB supplemental activities that you said you are familiar with. When we say supplemental activities, we are talking about all the surveys and tools that we asked about earlier.”*

1. What types of information are missing from your routine TB data collection systems in *[insert country]* that you need for planning and decision making?

[Prompt if didn’t mention: limitations in ability to use data - is there anything that makes it hard to use the data?]

*“Thank you. We will now focus our discussion on the TB supplemental activities.”*

2. Can you tell us what kind of important information was provided for your region by the **supplemental activities** that were conducted in *[insert country]*?

[Prompt with the list of activities implemented in country if needed.]

3. When supplemental activities are implemented, **are you typically informed of the results or recommendations**? If yes, how did you hear about the results or recommendations?

[Prompt with examples if needed: reports, email, workshops]

4. Have the **recommendations from supplemental activities impacted TB program planning or decision making in your region**? If yes, how? If no, why were the recommendations not useful?

This is a general question, but feel free to speak about specific tools if you’d like.

5. What could the National level team **do** to make the findings from supplemental activities **more useful for your region**?
6. Has the **implementation** of supplemental activities created any **challenges or opportunities** for you and your regional staff? If so, please describe. [Note: ONLY ASK if activities took place in their region.]
7. What would **you like the National level team to know** about your needs and experience with supplementary activities in your region?

Probe if not discussed:

- Implementation of the activity [*Only ask if aware of activities taking place in region*]
- Use of the results [*Only ask if aware of activities being implemented in country*]
- Are there TB programmatic areas that would benefit from additional tools?
- Do you see any benefit of conducting certain tools at subnational level? [*Only ask if aware of activities taking place in country*]

8. Is there anything else that you would like to tell us about the supplemental activities or data needs for program planning for the region?
