# Supplementary material for: Optimizing use of data and evidence-related tools to complement routine surveillance systems for tuberculosis program planning
Source: Front Tuberc. 2025 Jul 17;3:1622167. doi: 10.3389/ftubr.2025.1622167 (PMC13491421; doi:10.3389/ftubr.2025.1622167)
Supplement: Supplementary file 3 [file Table_3.docx]

# **Supplementary Material 3: Country use case discussion guide**

**TB Data Optimization Project: Country Use Case questions – [*COUNTRY*]**

**Introduction**

These questions are meant to be answered during a discussion with the NTP manager/head(s) of NTP and a group of 4-6 respondents. We encourage the NTP to include members of the TB Task Force or other working group that has substantial knowledge and experience with using data for TB program planning. The project team will arrange a time to meet with the NTP manager and other respondents using a tele-conferencing platform like Zoom to allow for discussion, follow-up questions, and clarifications.

The purpose of the discussion is for the project team to better understand how TB data activities have helped the NTP and partners to:

1. Estimate the burden of TB in their country
2. Understand and address specific gaps in the TB care cascade
3. Make both short- and long-term plans for the TB program

Each question will relate to one of the blue boxes in the figure below. This figure shows the TB-related data activities that have been conducted in [*country*] and may have been used to understand the item the blue box.

[Figure 1: Framework for use of data tools in different aspects of TB program evaluation and planning] (*Customized to be country-specific by showing only the activities that the country has conducted*)

^1^Shaded area = While routine data can provide some information on patients who presented to health facilities but were not diagnosed (e.g. screening data), TB program data often starts only with diagnoses or notifications.

^2^Shaded area = Unless data from a prevalence survey is available, countries may not have data on people with TB who did not access the health system to use in care cascade analyses.

^3^Tools from other sections = TB data tools listed under the “Estimate TB burden” and “TB care cascade” sections

We encourage the group of respondents to think about and discuss the questions as needed before meeting with the project staff.

**Questions for discussion**

*The first questions deal with estimation of the TB burden.*

**1a**. How do you **estimate the burden/level of drug-susceptible and drug-resistant TB in [*country*]**? When estimating the TB burden, how have you used different sources of data, including but not limited to the following sources?

Please consider two estimations of TB burden:

A) The true/actual burden of drug-susceptible and drug-resistant TB in your country
B) The figures used for target setting & planning

- Routine reporting – standard set of WHO indicators
- Routine reporting – additional indicators/analyses at country level
- WHO Global TB Report / Country profile
- Estimates from the Institutes for Health Metrics and Evaluation (IHME)
- Supplemental tools [*country*] has implemented:
- Any other surveys or assessments? (please specify the name of the survey/assessment)

**1b.** Which of these sources of data have been most critical for drug-susceptible and drug-resistant TB burden estimation? Why?

Are there any that you feel were less important or potentially not needed? Please explain.

**1c**. Are there other data or data tools/activities that would have been helpful to estimate the burden of DS or DR-TB?

*The next three questions will focus on specific gaps shown in the blue boxes of the TB Care Cascade below. When asking about each of these “gaps” we are referring to:* ***the number of people, their characteristics, and possible reasons for the gap****. Using the first blue box as an example, we would like to know: have efforts been made to understand or address the number of people with TB that do not access the health system, the characteristics of those that did not access the system, and/or the reasons for not accessing the system?*

[Figure 2: Framework for use of data tools to estimate different steps of the TB Care Cascade]

(*Customized to be country-specific by showing only the activities that the country has conducted*)

^1^Shaded area = While routine data can provide some information on patients who presented to health facilities but were not diagnosed (e.g. screening data), TB program data often starts only with diagnoses or notifications.

**2a**. The first gap we’ll discuss is accessing health care services. Have efforts been made to understand and/or address the gap in **people with TB who do not access health the health system**? If yes, how have you done this? If you used any of the following tools, please address the tool in your response.

- Routine reporting – standard set of WHO indicators
- Routine reporting – additional indicators/analyses at country level
- Supplemental tools [*country*] has implemented:
- Any other surveys or assessments? (please specify the name of the survey/assessment)

**2b**. Which of these activities have been most critical for understanding and/or addressing the gap in **people with TB who do not access the health system**? Why?

Are there any that you feel were less important or potentially not needed? Please explain.

**2c**. Are there other data or data tools that would have been helpful to understand and/or address the gap in **people with TB** **may not access the health system**? Please elaborate.

**3a**. The next gap we’ll discuss is **people with TB who sought health care, but were either not diagnosed or not notified to the NTP**. Have efforts been made to understand and/or address these gaps in **people with TB who sought health care, but were either not diagnosed or not notified to the NTP?** If yes, how have you done this? If you used any of the following tools, please address the tool in your response.

- Routine reporting – standard set of WHO indicators
- Routine reporting – additional indicators/analyses at country level
- Supplemental tools [*country*] has implemented:
- Any other surveys or assessments? (please specify the name of the survey/assessment)

**3b**. Which of these activities have been most critical for understanding and/or addressing the gap in **people with TB who sought health care but were either not diagnosed or not notified to the NTP**? Why?

Are there any that you feel were less important or potentially not needed? Please explain.

**3c**. Are there other data or data activities that would have been helpful to understand and/or address the gap in **people with TB who sought health care, but were either not diagnosed or not notified to the NTP**.? Please elaborate.

**4a.** The next gap we’ll discuss is under-reporting. Have efforts been made to understand and/or address the gap of people who are **diagnosed and notified to the NTP, but were not successfully treated**? **This includes people that did not initiate treatment and people that initiated but did not successfully complete treatment.** If yes, how have you done this? If you used any of the following tools, please address the tool in your response.

- Routine reporting – standard set of WHO indicators
- Routine reporting – additional indicators/analyses at country level
- Supplemental tools [*country*] has implemented:
- Any other surveys or assessments? (please specify the name of the survey/assessment)

**4b.** Which of these activities have been most critical for understanding and/or addressing the gap of people who were **diagnosed and notified to the NTP, but were not successfully treate**d? Why?

Are there any that you feel were less important or potentially not needed? Please explain.

**4c.** Are there other data or data activities that would have been helpful to understand and/or address the gap of people who were **diagnosed and notified to the NTP, but were not successfully treate**d?

*The last question deals with TB program planning. We are interested in three types of program planning:*

A. *National Strategic Plan (NSP) development*
B. *Global Fund applications*
C. *Routine/annual program planning*

**5a.** When planning for your national TB program, have you used the findings from the following activities? If yes, **how** have you used the findings from these tools? Please consider the three types of program planning mentioned above: preparing your National Strategic Plan, preparing your Global Fund application, and routine/annual program planning,

- Routine reporting – standard set of WHO indicators
- Routine reporting – additional indicators/analyses at country level
- Supplemental tools [*country*] has implemented:
- Any other surveys or assessments? (please specify the name of the survey/assessment)

**5b.** Which of these activities have been most critical for program planning? Why?

**5c.** Are there any that you feel were less important or potentially not needed? Please explain.

**5d.** Are there other data or data activities that would have been helpful for these planning activities? If yes, please detail/explain.
