# Supplementary material for: Optimizing use of data and evidence-related tools to complement routine surveillance systems for tuberculosis program planning
Source: Front Tuberc. 2025 Jul 17;3:1622167. doi: 10.3389/ftubr.2025.1622167 (PMC13491421; doi:10.3389/ftubr.2025.1622167)
Supplement: Supplementary file 4 [file Table_4.docx]

# **Supplementary Material 4: National TB program survey questions**

**TB Data Optimization**

**Online Survey for NTP Managers/Data Officers**

**Introduction:**

- This project, “Optimizing TB analytics and evidence tools to improve data use in TB programmatic planning” aims to develop recommendations to improve the collection and use of TB data for program planning and decision making.
- The project focuses on the use of **supplementary TB data activities** such as TB Prevalence Surveys, TB Drug Resistance Surveys, Patient Cost Surveys, Patient Pathway Analysis, Epidemiological Reviews, and other activities that are conducted occasionally to supplement routine TB data collection and analysis.
- We are requesting **one survey per country**. The ideal respondent is someone in the National TB Program who is very familiar with the supplemental TB data activities that have been conducted in your country to complete this survey. For example, someone who:
  - Planned and/or implemented some of the supplemental TB data activities in your country
  - Used the results from the supplemental TB data activities in your country
  - Participated in the development of the last TB National Strategic Plan
- The respondent is welcome to solicit input from other NTP staff (e.g. M&E or data officer, program managers/coordinators, research team) that are familiar with and/or have historical knowledge of the supplementary TB data activities referenced in the survey, however it is easiest to complete the online survey at one time. We suggest reviewing the paper survey first and using it to solicit additional input needed prior to starting the online survey.
- Taking part in this survey is voluntary. Choose the “prefer not to answer option” if you do not feel comfortable answering the question.
- This survey is an opportunity for your country’s TB program to provide feedback to global partners and funders and potentially influence global recommendations.
- There is no cost to you for participating, nor will you be paid to participate in the project. We thank you for the time you took to participate in this activity.

The project team will try to answer all your questions. If you have questions about the study, please contact Rachel Fiorillo at rfiorillo@cdcfoundation.org.

| **5-digit Unique Project ID:**  *(Required)*  *Provided in email* | __ __ __ __ __ |
| --- | --- |

**CONSENT:**

| Do you consent to participating in this survey?  *(Required)* | ☐ Yes (proceed to questionnaire)  ☐ No (STOP HERE, END OF SURVEY) |
| --- | --- |

A. **DEMOGRAPHICS**

| **1** | WHO Region  *(Required)*  (*Select one*) | ☐ African Region (AFRO)  ☐ Region of the Americas (AMRO)  ☐ Eastern Mediterranean Region (EMRO)  ☐ European Region (EURO)  ☐ South-East Asia Region (SEARO)  ☐ Western Pacific Region (WPRO) |
| --- | --- | --- |
| **2** | Total years working in TB program, including national program and subnational levels (provincial, regional etc.)  *(Required)*  (If multiple people helping to complete this survey, please respond for the primary respondent) | _________ years  ☐ Prefer not to answer |
| **3** | What is your current position?  *(Required)*  (If multiple people helping to complete this survey, please respond for the primary respondent) | ☐ NTP Manager/Head of NTP  ☐ Acting NTP Manager/Head of NTP  ☐ Head of M&E  ☐ M&E Officer/Data Officer  ☐ Head of Research  ☐ Research Officer/Researcher  ☐ Other (specify): ____________  ☐ Prefer not to answer |

B. **FAMILIARITY WITH SUPPLEMENTAL TB DATA ACTIVITIES**

| **1** | **For the following activities, please indicate if your country has implemented the activity.** | |
| --- | --- | --- |
| **a** | **National TB Prevalence Survey**  *Large national survey where community members are screened for TB to estimate the true burden of TB.* | ☐ Yes, multiple times  ☐ Yes, one time  ☐ My country has not implemented it yet, but is currently planning this activity  ☐ No, my country has not implemented this activity  ☐ Heard of the activity but don’t know if my country has implemented it  ☐ Never heard of this activity  ☐ Prefer not to answer |
| **b** | **TB Drug Resistance Survey**  *Nationwide survey where sputum samples are collected from pulmonary TB patients and tested for resistance to determine the burden and pattern of drug-resistant TB.* | *This question will have the same response options as above.* |
| **c** | **TB Inventory Study**  *National study where TB patient records from the national surveillance system are linked with other available case-based databases (e.g. laboratory registers) to examine the level of underreporting.* | *This question will have the same response options as above.* |
| **d** | **Private Sector Drug Sales Analysis**  *An analytic approach to estimate the volume of TB patients treated by private sector providers using non-NTP drugs.* | *This question will have the same response options as above.* |
| **e** | **TB Patient Cost Survey/Catastrophic Cost Survey**  *A nationwide survey among TB patients conducted at selected health facilities to estimate and understand the costs incurred by TB patients.* | *This question will have the same response options as above.* |
| **f** | **TB Service Delivery Costing Study (Value TB)**  *A tool that estimates the cost of delivering TB interventions and services at the facility level.* | *This question will have the same response options as above.* |
| **g** | **One Health Tool for TB Budgeting**  *A costing tool used to estimate resources required to implement the TB national strategic plan.* | *This question will have the same response options as above.* |
| **h** | **People Centered Framework**  *A framework used for the development of the national strategic plan; it consolidates many sources of data to look at potential gaps in the TB care cascade.* | *This question will have the same response options as above.* |
| **i** | **TB Care Cascade Analysis**  *An analytic approach to assess the TB continuum of care and outcomes for all the estimated annual TB patients in the country and illustrate where losses occur.* | *This question will have the same response options as above.* |
| **j** | **MATCH Analysis**  *An analytic approach which uses subnational level spatial and program data to identify gaps in TB service delivery within subnational areas.* | *This question will have the same response options as above.* |
| **k** | **Patient Pathway Analysis**  *A tool that uses existing data to look at patient care-seeking practices and how they align with the availability of TB diagnostic and treatment services.* | *This question will have the same response options as above.* |
| **l** | **Diagnostic Network Optimization**  *An analytic approach to look at how diagnostic services are organized in a country to inform the optimal location of TB diagnostic tools like GeneXpert.* | *This question will have the same response options as above.* |
| **m** | **TB Diagnostic Network Assessment**  *A tool to assess the functionality of a national TB diagnostic network from the perspective of its ability to meet the needs of the TB national strategic plan.* | *This question will have the same response options as above.* |
| **n** | **TB Epidemiological Review, including Standards and Benchmarks**  *A review of the routine TB surveillance system and TB data in the country (national and subnational levels) to look at the trend of key TB indicators to understand the epidemic ahead of strategic planning.* | *This question will have the same response options as above.* |
| **o** | **Quality of TB Services Assessment**  *A survey conducted at a nationwide sample of health facilities where TB staff and patients are surveyed to assess the quality of TB services in the health facility.* | *This question will have the same response options as above.* |
| **p** | **Epidemiological Modelling**  *A data modelling activity conducted at the national level to better understand the potential impact of interventions on disease burden and program costs.* | *This question will have the same response options as above.* |
| **q** | **Screen-TB**  *A web-based tool used to compare different TB screening strategies and assess the expected cost and effectiveness of potential approaches, and their risks and benefits.* | *This question will have the same response options as above.* |

| **2** | **For the supplemental TB data activities your country has implemented, please indicate how you were involved.**  (If multiple people helping to complete this survey, please respond for the primary respondent) | |
| --- | --- | --- |
| *The first supplemental TB data activity you indicated your country has implemented (e.g. TB Prevalence survey, TB Drug Resistance survey, etc.) will be inserted here.*  (*Select all that apply*) | | ☐ Involved in planning  ☐ Involved in implementing  ☐ Have seen/heard/used the results  ☐ Not involved and do not know the results  ☐ Prefer not to answer |
| *This question will be repeated for each supplemental TB data activity your country has implemented.*  (*Select all that apply*) | | ☐ Involved in planning  ☐ Involved in implementing  ☐ Have seen/heard/used the results  ☐ Not involved and do not know the results  ☐ Prefer not to answer |

C. **QUESTIONS**

| **1** | Which of the following do you consider the top challenges in the generation, analysis, and use of TB data collected by routine data systems in your country?  **Routine data systems: a country’s TB/infectious disease surveillance system or health management information system.*  *(Select up to 5)* | ☐ Some key data are not collected by routine systems  ☐ Availability of real-time data  ☐ Paper-based system  ☐ Aggregate data, not case-based  ☐ Data quality  ☐ Data flow is only from lower levels to higher levels  ☐ Limited use of the data at national level  ☐ Limited use of the data at lower levels  ☐ Limited data analysis capacity at national level  ☐ Limited data analysis capacity at lower levels  ☐ Other (specify): ______________  ☐ Prefer not to answer |
| --- | --- | --- |
| **2** | In general, who **most often** suggests/proposes implementation of a supplementary activity?  (*Select one*) | ☐ The National TB Program/Ministry of Health  ☐ In-country TB technical working group  ☐ In-country partners (e.g. local academic institutions, local NGOs)  ☐ International technical partners (e.g. WHO, KNCV, CDC, etc.)  ☐ Funding partners (e.g. Global Fund, USAID, Bill & Melinda Gates Foundation, etc.)  ☐ Other (specify): ______________  ☐ Prefer not to answer |
| **3** | In general, which of the following is the **strongest** motivating factor for your country to implement supplemental activities?  (*Select one*) | ☐ Country’s need for more data or evidence  ☐ Measure progress towards Global TB strategies  ☐ Request by donor  ☐ Recommended by partners  ☐ Availability of funding  ☐ Other (specify): ______________  ☐ Prefer not to answer |
| **4** | Who typically makes the **final** decision to implement supplemental activities in your country?  (*Select one*) | ☐ The National TB Program/Ministry of Health  ☐ In-country partners (e.g. local academic institutions, local NGOs)  ☐ International technical partners (e.g. WHO, KNCV, CDC, etc.)  ☐ Funding partners (e.g. Global Fund, USAID, Bill & Melinda Gates Foundation, etc.)  ☐ Other (specify): ______________  ☐ Prefer not to answer |
| **5** | Have you ever felt pressure from external partners or funding agencies to implement a tool that was not perceived as a priority for the NTP? | ☐ Yes, this has happened multiple times  ☐ Yes, this has happened one time  ☐ No, this hasn’t happened  ☐ Prefer not to answer |
| **5a** | If selected either ‘yes’ response to question 5: Which activity(ies) did you feel pressured to conduct that were not a priority for the NTP?  *(Select multiple)* | ☐ (A list of supplemental TB data activities your country has implemented will be listed)  ☐ Other (specify): ______________  ☐ Prefer not to answer |
| **6** | What are the **most** significant challenges you have encountered with planning and implementing supplemental activities?  *(Select up to 5; if selected ‘none of the above’ do not select any other option)* | ☐ Insufficient financial resources/funding  ☐ Limited technical capacity of NTP staff to plan/implement activity  ☐ Insufficient number of NTP staff or time to plan/implement activity  ☐ Insufficient technical capacity to analyze data and/or write report  ☐ Insufficient staff time to analyze data and/or write report  ☐ Data availability for activities that need existing data  ☐ Lack of coordination between partners  ☐ Procurement challenges delay implementation  ☐ Delayed receipt of results hinders their use  ☐ Increases workload of TB program staff  ☐ Impact on routine program activities because staff are working on supplemental activity  ☐ Other (specify): ______________  ☐ None of the above  ☐ Prefer not to answer |
| **7** | Which are the **most** significant opportunities or benefits you have experienced from implementing supplemental activities?  *(Select up to 5)* | ☐ Financial support from partners for the activities  ☐ Technical support from partners for the activities  ☐ Government commitment  ☐ Opportunity to work with partners and funders  ☐ Opportunity to build capacity of NTP staff  ☐ Activities provide timely information during the program planning cycle  ☐ Activities provide structure and/or equipment for routine TB activities  ☐ Activities provide research opportunities  ☐ Activities fuel advocacy for TB support & TB education  ☐ Other (specify): ______________  ☐ None of the above  ☐ Prefer not to answer |

| **8** | **For each of the following activities that have been implemented in your country, can you tell us how important the findings were to helping you understand gaps in your TB care cascade?** | |
| --- | --- | --- |
| *This question will be asked for each of the supplemental TB data activities your country has implemented.* | | ☐ Very important/helpful ☐ Somewhat important/helpful ☐ Not important/helpful  ☐ Prefer not to answer |

| **9** | **For each of the following activities that have been implemented in your country, can you tell us how important the findings were to helping you routine programmatic planning and forecasting?** | |
| --- | --- | --- |
| *This question will be asked for each of the supplemental TB data activities your country has implemented.* | | ☐ Very important/helpful ☐ Somewhat important/helpful ☐ Not important/helpful  ☐ Prefer not to answer |

| **10** | **For each of the following activities that have been implemented in your country, can you tell us how important the findings were to helping you to monitor progress towards targets?** | |
| --- | --- | --- |
| *This question will be asked for each of the supplemental TB data activities your country has implemented.* | | ☐ Very important/helpful ☐ Somewhat important/helpful ☐ Not important/helpful  ☐ Prefer not to answer |

| **11** | **For each of the following activities that have been implemented in your country, can you tell us how important the findings were to helping you develop your TB National Strategic Plan?** | |
| --- | --- | --- |
| *This question will be asked for each of the supplemental TB data activities your country has implemented.* | | ☐ Very important/helpful ☐ Somewhat important/helpful ☐ Not important/helpful ☐ Prefer not to answer |

| **12** | **For each of the following activities that have been implemented in your country, can you tell us how important the findings were to helping you develop funding applications (e.g. Global Fund proposal)?** | |
| --- | --- | --- |
| *This question will be asked for each of the supplemental TB data activities your country has implemented.* | | ☐ Very important/helpful ☐ Somewhat important/helpful ☐ Not important/helpful ☐ Prefer not to answer |

| **13** | **For each of the following activities that have been implemented in your country, can you tell us how important the findings were to impacting your country’s guidelines and/or policies?** | |
| --- | --- | --- |
| *This question will be asked for each of the supplemental TB data activities your country has implemented.* | | ☐ Very important/helpful ☐ Somewhat important/helpful ☐ Not important/helpful  ☐ Prefer not to answer |

| **14** | How are the results from supplemental data activities **most often** used?  (*Select one*) | ☐ Mostly internal use (e.g. reporting to NTP or MoH, NSP development)  ☐ Mostly external use (e.g. reporting to outside partners or funders, development of external funding proposals)  ☐ Equal mix of internal and external use ☐ None of the above (please explain):__________________________  ☐ Prefer not to answer |
| --- | --- | --- |
| **15** | If you received a large grant for TB data activities that you could spend as you choose, how much would you allocate to strengthening routine data systems, with the rest going towards supplemental data activities?  (*Select one*) | ☐ 76-100% towards strengthening routine data systems  ☐ 51-75% towards strengthening routine data systems  ☐ 26-50% towards strengthening routine data systems  ☐ 0-25% towards strengthening routine data systems  ☐ Prefer not to answer |

| **16** | **For the following activities, please indicate whether (a) you think the activity was worth the investment and (b) you would want to implement it again.** | a. Please indicate whether you think the outcomes of the following activities were worth the required investment of resources (money, staff, time, etc.)? | b. Do you think it would be important for your country to implement the activity again? | c. For each of the following activities that you said you **would repeat**, what (if anything) would you do differently the next time you implement it? | d. If no to implementing the activity again, why not? | e. If no to implementing the activity again, is there another way you would collect or analyze similar data? |
| --- | --- | --- | --- | --- | --- | --- |
| *This question will be asked for each of the supplemental TB data activities your country has implemented.* | | ☐ Yes  ☐ No  ☐ Unsure  ☐ Prefer not to answer | ☐ Yes  ☐ No  ☐ Unsure  ☐ Prefer not to answer |  |  |  |

| **17** | Please indicate which of the following activities you think would be important for your country to implement in the future.  *(Select multiple)*  *(If selected ‘None’, do not select other options)* | ☐ (The TB data activities that your country has **not yet** implemented will be listed here)  ☐ Other (specify): _______  ☐ None  ☐ Prefer not to answer |
| --- | --- | --- |

| **18** | **Please indicate how much technical support you received from partners for the following activities. Consider support needed for all the phases of an activity, including: planning, implementation, data analysis, reporting and dissemination, and implementation of recommendations.** | | |
| --- | --- | --- | --- |
| **List of activities** | | How much technical support did you receive from external partners for this activity? | If inadequate support received: Which of the following areas needed more technical support?  (*Select multiple*) |
| *This question will be asked for each of the supplemental TB data activities your country has implemented.* | | ☐ Little or no support needed  ☐ Support needed and adequate support received  ☐ Support needed but inadequate support received  ☐ Unsure  ☐ Prefer not to answer | ☐ Planning  ☐ Implementation  ☐ Data analysis  ☐ Reporting and dissemination  ☐ Implementation of recommendations  ☐ Translation into policy  ☐ Other (specify):_________________  ☐ Prefer not to answer |

| **19** | **Please indicate how much funding support you received from partners for the following activities. Consider support needed for all the phases of an activity, including: planning, implementation, data analysis, reporting and dissemination, and implementation of recommendations.** | | |
| --- | --- | --- | --- |
| **List of activities** | | How much funding support did you receive from external partners for this activity? | If inadequate support received: Which of the following areas needed more funding support?  (*Select multiple*) |
| *This question will be asked for each of the supplemental TB data activities your country has implemented.* | | ☐ Little or no support needed  ☐ Support needed and adequate support received  ☐ Support needed but inadequate support received  ☐ Unsure  ☐ Prefer not to answer | ☐ Planning  ☐ Implementation  ☐ Data analysis  ☐ Reporting and dissemination  ☐ Implementation of recommendations  ☐ Other (specify):_________________  ☐ Prefer not to answer |

| **20** | Considering your country’s routine data and the supplemental activities that have been implemented, what important information is **STILL needed or missing** for program planning and decision making? |  |
| --- | --- | --- |

D. **ADDITIONAL COMMENTS**

| Please share any additional comments or feedback about your country’s experience with supplemental TB data activities: |  |
| --- | --- |

**END OF SURVEY. THANK YOU!**
