# Supplementary material for: Optimizing use of data and evidence-related tools to complement routine surveillance systems for tuberculosis program planning
Source: Front Tuberc. 2025 Jul 17;3:1622167. doi: 10.3389/ftubr.2025.1622167 (PMC13491421; doi:10.3389/ftubr.2025.1622167)
Supplement: Supplementary file 5 [file Table_5.doc]

# **Supplementary Material 5: List of countries that completed the National TB Program (NTP) survey**

| **Number** | **NTP Survey Country** | **World Bank Income Group** | **Region** |
| --- | --- | --- | --- |
| 1 | Belarus | Upper middle income | Europe |
| 2 | Bhutan | Lower middle income | Asia |
| 3 | Botswana | Upper middle income | Africa |
| 4 | Brazil | Upper middle income | South America |
| 5 | Burkina Faso | Low income | Africa |
| 6 | Cambodia | Lower middle income | Asia |
| 7 | Cameroon | Lower middle income | Africa |
| 8 | China | Upper middle income | Asia |
| 9 | DR Congo | Low income | Africa |
| 10 | Egypt | Lower middle income | Africa |
| 11 | Eswatini | Lower middle income | Africa |
| 12 | Ethiopia | Low income | Africa |
| 13 | Fiji | Upper middle income | Oceania |
| 14 | Georgia | Upper middle income | Asia |
| 15 | Ghana | Lower middle income | Africa |
| 16 | India | Lower middle income | Asia |
| 17 | Kenya | Lower middle income | Africa |
| 18 | Lao PDR | Lower middle income | Asia |
| 19 | Lesotho | Lower middle income | Africa |
| 20 | Liberia | Low income | Africa |
| 21 | Malawi | Low income | Africa |
| 22 | Republic of Moldova | Upper middle income | Europe |
| 23 | Mongolia | Lower middle income | Asia |
| 24 | Mozambique | Low income | Africa |
| 25 | Namibia | Upper middle income | Africa |
| 26 | Nepal | Lower middle income | Asia |
| 27 | Nigeria | Lower middle income | Africa |
| 28 | Pakistan | Lower middle income | Asia |
| 29 | Papua New Guinea | Lower middle income | Oceania |
| 30 | Peru | Upper middle income | South America |
| 31 | Philippines | Lower middle income | Asia |
| 32 | Rwanda | Low income | Africa |
| 33 | Sierra Leone | Low income | Africa |
| 34 | Solomon Islands | Lower middle income | Oceania |
| 35 | South Africa | Upper middle income | Africa |
| 36 | Sri Lanka | Lower middle income | Asia |
| 37 | Tajikistan | Lower middle income | Asia |
| 38 | Tanzania | Lower middle income | Africa |
| 39 | Uganda | Low income | Africa |
| 40 | Vietnam | Lower middle income | Asia |
| 41 | Zambia | Low income | Africa |
| 42 | Zimbabwe | Lower middle income | Africa |
